# Revision of series *Gravesiana* (*Adiantum* L.) based on morphological characteristics, spores and phylogenetic analyses

**DOI:** 10.1371/journal.pone.0172729

**Published:** 2017-04-05

**Authors:** Ai-Hua Wang, Fa-Guo Wang, Wan-Wan Zhang, Xiao-Dong Ma, Xu-Wen Li, Qi-Fei Yi, Dong-Lin Li, Lei Duan, Yue-Hong Yan, Fu-Wu Xing

**Affiliations:** 1 Key Laboratory of Plant Resources Conservation and Sustainable Utilization, South China Botanical Garden, Chinese Academy of Sciences, Guangzhou, China; 2 Key Laboratory of Environment Change and Resources Use in Beibu Gulf (Guangxi Teachers Education University), Ministry of Education, Nanning, China; 3 University of Chinese Academy of Sciences, Beijing, China; 4 Guangzhou Institute of Forestry and Landscape Architecture, Guangzhou, China; 5 Shanghai Chenshan Plant Science Research Center, Chinese Academy of Sciences, Shanghai, China; The National Orchid Conservation Center of China; The Orchid Conservation & Research Center of Shenzhen, CHINA

## Abstract

Since the adoption of some ambiguous and quantitative characters in *Flora Republicae Popularis Sinicae 3(1)*, species identifications of the series *Gravesiana* have been in disarray, requiring clarification. Two hundred and fifty-nine individuals from 47 different populations were collected for the estimation of morphological characters and phylogenetic analyses. Spores of 26 populations were observed through scanning electron microscope. Our results were different from those of previous research: (1) six identifiable species, rather than five species observed previously, were confirmed in the series *Gravesiana*, they are *A*. *gravesii*, *A*. *juxtapositum*, *A*. *mariesii*, *A*. *dentatum*, *A*. *longzhouensis* and *A*. *obovatum*, of which the latter three are newly recognized species. (2) Thirteen characters were measured and estimated through the program Mesquite v. 2.71. The character whether the pinna stalks were 1/3-1/2 times longer than the pinna was used to distinguish *A*. *gravesii* and *A*. *lianxianense* previously and was found to be unreliable here, whereas such characters as the height of the plant (H), pinna aligned forms (FP), number of pinna (NP), pinna margin (M), number of veins flabellate at base (NV), sori number and shape per pinna (NSS), pinna texture (T), and powder-covered or not on the abaxial surface of the pinna (P) are estimated to be stable and reliable characters useful for identification. Descriptions of new species and their retrieve keys are also listed. (3) Surface ornamentations and spore sizes are helpful for us to distinguish species in series *Gravesiana*.

## Introduction

Pteridaceae is a large family containing approximately 50 genera and 950 species, with most of them existing in tropical and arid regions [1]. There are 20 genera and 233 species (89 endemic, one introduced) in five subfamilies distributed in China [1]. *Adiantum* L. is a large genus in the Pteridaceae, with more than 200 species worldwide distributed except in extremely cold or dry regions [1, 2]. In China, there are approximately 34 species, 16 of which are endemic [1]. Lin (1980) [3] classified them into seven series: *Caudata*, *Flabellulata*, *Gravesiana*, *Pedata*, *Reniformia*, *Veneri-Capilliformia* and *Venusta*. However, it was only partially consistent with the phylogenetic results because the series *Gravesiana* was nested within one subgroup of the series *Caudata* [2]. Series *Gravesiana*, containing 5 species, namely *A*. *chienii* Ching, *A*. *gravesii* Hance, *A*. *juxtapositum* Ching, *A*. *lianxianense* Ching & Y.X. Lin and *A*. *mariesii* Baker [4], are endemic, and they mainly exist in Karst landforms or Danxia landforms in China. However, previous identifications of these five species have been ambiguous because of incomplete sampling [3, 5, 6, 7, 8], and some quantitative characters were used in *Flora Republicae Popularis Sinicae* [4].

**Fig 1:**
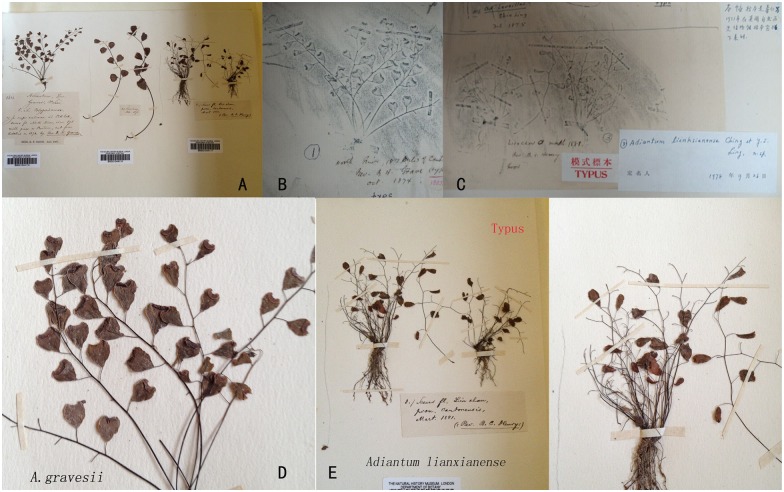
The type specimens of *A*. *gravesii* and *A*. *lianxianense*. A: the whole view. B and C: the tracings of the type specimen of *A*. *gravesii* and *A*. *lianxianense*. D and E: the enlarged type specimens of *A*. *gravesii* and *A*. *lianxianense*.

There are few collected specimens of *A*. *lianxianense*, and its relative studies are also rare, possibly due to the low-quality pictures of its typus (Fig 1C) when it was first published by Lin (1980) [3]. So it is hard to identify *A*. *lianxianense* precisely. According to Lin (1980) [3] and Ching [4], *A*. *gravesii* is similar to *A*. *lianxianense* but its plant texture is much harder and stronger than *A*. *lianxianense*. Besides, whether the pinna stalks were 1/3-1/2 times longer than the pinna is the main difference between *A*. *gravesii* and *A*. *lianxianense*. *A*. *juxtapositum* is considered as an endemic species in Xianxia Ling, Fujian province [4]. However, Yan (2011, 2012) [9, 10] found it in some Danxia landforms of Hunan, Jiangxi and Guangdong. *A*. *chienii* is an endemic species of the North River, Guangdong province in China. It is similar to *A*. *juxtapositum* but can be easily distinguished by its one (rarely two) sori per pinna, while *A*. *juxtapositum* has three to many sori per pinna [1, 7, 8]. Unfortunately, few specimens of *A*. *chienii* have been collected and kept except for its typus. The morphological characters of these five species are shown in Table 1.

**Table 1:** Main morphological characters of the five species in series *Gravesiana* according to Shing and Wu (1990).

Species	Main characters
*A*. *chienii*	Plants erect, larger and much stronger. Pinna 5 or more, obdeltoid. Indusium 1 per pinna, terminal truncate.
*A*. *gravesii*	Plants erect, more than 3 cm high. Pinna 5 or more, broad-fanshaped, pinna stalk length less than 1/5 times of pinna. Indusium 1 per pinna, transversally linear, terminal concave.
*A*. *juxtapositum*	Plants erect, larger and much stronger. Pinna 5 or more, broad-fanshaped to subround. Indusium often 3–4 per pinna, terminal truncate.
*A*. *lianxianense*	Plants erect, slender, more than 3 cm high. Pinna narrow-fanshaped, membranous, pinna stalk can be 1/3-1/2 timers long of pinna. Indusium 1 per pinna.
*A*. *mariesii*	Mattae, short, 1.5–3 cm. pinna 3–5, small and sub-round. Indusium 1 per pinna, orbicular.

**Fig 2:**
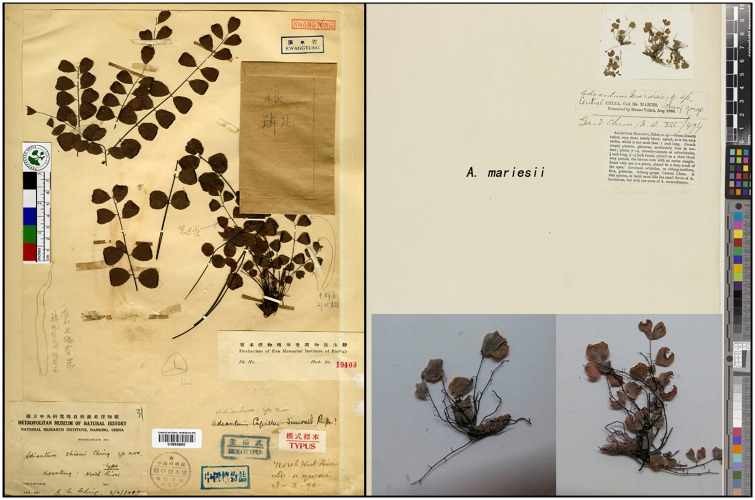
The type specimens of *A*. *chienii* and *A*. *mariesii*. Left: *A*. *chienii*; right: *A*. *mariesii*.

**Fig 3:**
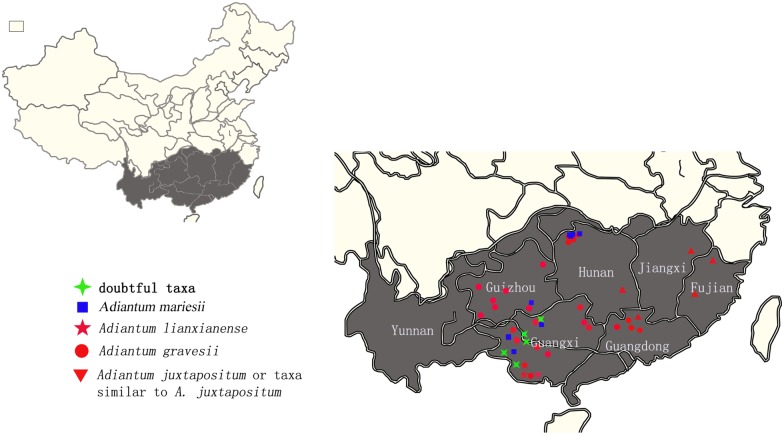
The distribution and collection sites of different populations in the series *Gravesiana*.

*A*. *chienii*, *A*. *gravesii* and *A*. *juxtapositum* share a common character—the length of the pinna stalks is less than 1/5 times that of the pinna. However, when we examined each of their specimens (including typus Figs 1 and 2), pinna stalks from different parts of a lamina were of different length; some reached up to 1/5 of the pinna while others did not. The biggest distinction of these three species is that *A*. *gravesii* is alternipinnate and each pinna has only 1 or 2 nephroid or transversally linear sori with a sinus in the middle, whereas *A*. *chienii* and *A*. *juxtapositum* possess opposite pinna and each pinna has 1-to-many sori with apex truncate. However, some new individuals (doubtful taxa, short for DT) with alternating pinna and 3-to-many kidney-shaped sori per pinna were found during our field investigation. We found some individuals from different populations with similar new characters never encountered before (DT). In addition, many individuals have highly diverse characters in one population. Hence, do these variable individuals belong to one species or to different species? To elucidate the identification of these five species, we investigated a total of 47 populations from Guangxi, Guizhou, Guangdong, Hunan, Jiangxi and Fujian provinces of China (Fig 3**)** and collected 259 individuals. Such characters as the height of the plant, leaf stalk, pinna stalk, pinna size and shape, veins, sori number per pinna, and scales were measured before DNA extraction. Combined analyses of microscopic spore observations, morphological and molecular data will be carried out to resolve the phylogenetic relationship of the series *Gravesiana* here.

## Materials and methods

259 individuals from 49 populations were collected and silica-gel-dried for DNA extraction, of which 22 populations were from *A*. *gravesii*, 4 populations were from *A*. *juxtapositum*, 2 populations were from *A*. *lianxianense*, 8 populations were from *A*. *mariesii*, and the other 13 populations were doubtful taxa (Fig 3)(no individuals of *A*. *chienii* were collected?). Six plastid genes (*atpA*, *atpB*, *rbcL*, *trnL-F*, *trnS* and *matK*) of 76 individuals from 42 populations were amplified and sequenced to generate a phylogenetic tree of the series *Gravesiana*. To construct the phylogenetic tree of Chinese *Adiantum*, five chloroplast genes (*atpA*, *atpB*, *rbcL*, *trnL-F* and *trnS*) of *A*. *nelumboides* X.C. Zhang (2 accessions), *A*. *reniforme* L. (11 accessions), *A*. *malesianum* Ghatak, and *A*. *induratum* Christ were used [11], and another 33 *Adiantum* species were downloaded from GenBank. *Vittaria flexuosa* Fée was selected as outgroup. Details on the collection of the 78 individuals of the series *Gravesiana* including collection locality and date, longitude, latitude and altitude are shown in S1 Table, and the information for all other taxa are listed in S2 Table.

### DNA sequencing and phylogenetic analyses

Total DNA was extracted from silica-gel-dried leaf materials using a modified CTAB DNA extraction protocol [12]. Six pairs of primers “ESATPF412F and ESTRNR46F”, “ESATB172F and ESATPE45R”, “1F and 1379R”, “f and pl”, “trnS and rps4.5”, “Adn matK fIHS* and FER matK rAGK” were used to amplify the chloroplast gene regions *atpA*, *atpB*, *rbcL*, *trnL-F*, *rps4-trnS*, and *matK*, respectively [13, 14, 15, 16, 17, 18, 19, 20]. PCR reactions were performed in 30 μL reaction volumes, including 1.0–2.4 μL of each primer (5p), 17–60 ng sample DNA, 1.5 U of Taq DNA polymerase, 10 × buffer (including Mg^2+^), 0.25 mmol·L^-1^ dNTP, and ultrapure water. The PCR products were purified and sequenced with an ABI 3730XL by Majorbio Company.

The sequences were assembled with Sequencher v. 4.14, aligned using the program Clustal X v. 2.0 [21] and then edited manually through Bioedit v.7.1.3 [22]. Phylogenetic trees of each marker and the combined markers (*atpA*, *atpB*, *rbcL*, *trnL-F*, *rps4-trnS* and *matK*) were constructed using maximum parsimony (MP) and Bayesian Markov chain Monte Carlo inference (BI). The maximum parsimony analyses were performed with PAUP* 4.0b10 [23], treating gaps as missing data and using the heuristic search options with 1000 random replicates and tree-bisection-reconnection (TBR) branch swapping. All characteristics were unordered and equally weighted. Through MrModeltest2 v. 2.3 [24], GTR+I+G was selected as the best fit molecular evolution model for the MP and Bayesian analyses. For Bayesian inference, trees were generated for 1,000,000 generations with sampling every 100 generations. Four chains were used with a random initial tree. For each of the individual data partitions and the combined dataset, the first 2500 sample trees were discarded as burn-in to ensure that the chains reached stationarity. Nodes receiving bootstrap support (BS) of < 70% in the MP analyses or PP of < 0.95 in the BI analyses were not considered to be well supported.

### Morphological characters analyses

Thirteen characters of the 78 individuals including the height of the plant (H), the length of pinna stalk (LR), pinna aligned forms (FP), number of pinna (NP), pinna size (S) and shape (SP), pinna margin (M), number of veins flabellate at base (NV), veins tendency near upper margin (TV), sori number and shape per pinna (NSS), pinna texture (T), powder-covered or not on the abaxial surface of the pinna (P), and scale shape (SS) were measured before DNA extraction. The morphologic data were maintained in a spreadsheet and then compiled into a digital matrix. Then, we mapped them onto the phylogenetic tree of the series *Gravesiana* constructed with six combined chloroplast genes, *atpA*, *atpB*, *rbcL*, *trnL-F*, *rps4-trnS* and *matK*, using the program Mesquite version 2.71 [25] under maximum parsimony. Original measured values of the morphological characters were presented in S3 Table. The classifications of the characters mapped are listed below:

1. Height of the plant (H): (0) H ≤5 cm, (1) 5 cm<H≤13 cm, (2) H>13 cm
2. Length of the pinna stalk (LR): (0) 0 mm<LR≤1 mm, (1) 0 mm<LR≤3 mm, (2) 0 mm<LR≤5 mm
3. Pinna aligned forms (FP): (0) alternate, (1) opposite
4. Number of pinna (NP): (0) 1<NP≤3, (1) 3<NP≤6, (2) 6<NP≤9, (3) NP>9;
5. Pinna size (S: length × width, unites/cm): (0) 2.0–6.0 × 2.0–5.0, (1) 6.0–15.0 × 5.0–17.0, (2) 6.0–15.0 × 2.0–5.0
6. Pinna shape (SP): (0) obovate, (1) obdeltoid, (2) subround, (3) others
7. Pinna margin (M): (0) entire, (1) dentate
8. Number of veins flabellate at base (NV): (0) NV≤4(5), (1) NV>4(5)
9. Vein tendency near upper margin (TV): (0) straight up to terminal, (1) curve closed to margin
10. Sori number and shape per pinna (NSS): (0) 1, orbicular; (1) 1, reniform; (2) 1 to many, reniform; (3) 1 to many, transversally linear and truncate at false indusia terminal; (4) 1, shape same as (3)
11. Pinna texture (T): (0) membranous, (1) coriaceous
12. Powder-covered or not on abaxial surface of pinna (P): (0) no, (1) yes

### SEM observation

Spores from 26 different populations of series *Gravesiana* were observed through scanning electron microscopy. Mature spores from the same population were dispersed on stubs directly after being collected and marked with a unique population ID. The spores were gold-coated in a JFC-1600 Auto Fine Coater and observed using a JEOL JSM-6360LV Scanning Electron Microscope at 25 kV at the South China Botanical Garden, Chinese Academy of Sciences. The spore mean sizes were measured by Smile View software (20 spores per population). The descriptive terminology in *Spores of Polypodiales (Filicales) from China* [26] and *Plant identification terminology*: *An illustrated glossary* [27] was followed.

### Nomenclature

The electronic version of this article in Portable Document Format (PDF) in a work with an ISSN or ISBN will represent a published work according to the International Code of Nomenclature for algae, fungi, and plants, and hence the new names contained in the electronic publication of a PLOS ONE article are effectively published under that Code from the electronic edition alone, so there is no need to provide printed copies.

IPNI LSIDs (Life Science Identifiers) for new species herein have been resolved and can be available at http://ipni.org, once the paper is published. The online version of this work is archived and available from the following digital repositories: PubMed Central, LOCKSS.

## Results

### Phylogenetic analyses

**Fig 4:**
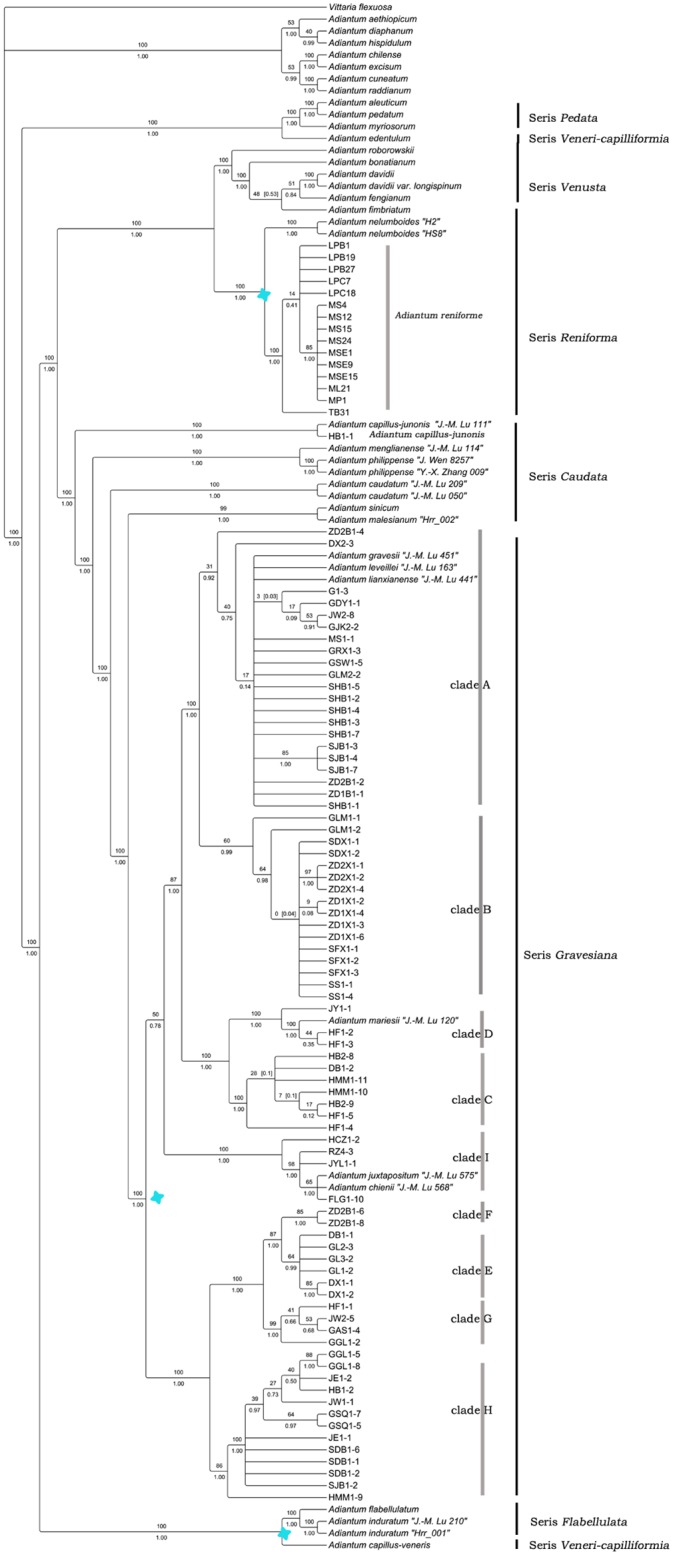
Strict consensus tree of five maximally parsimonious trees for Chinense *Adiantum*, generating from *atpA*, *atpB*, *rbcL*, *trnL-F* and *rps4-trnS* sequences (tree length = 5,052 steps, CI = 0.6033, RI = 0.9198). The bootstrap values were shown above the lines, and the Bayesian posterior probabilities were shown below the lines. Series classifications of Lin (1980) about *Adiantum* were labeled in columns. For G1-3, GDY1-1, JW2-8, GJK2-2, …., front alphabets are the short names of different populations of taxa, and the latter numbers represent single individuals as shown in S1 Table.

The topologies derived from the analyses of single dataset and the combined dataset were congruent; thus, we adopted the topology from the combined dataset here. The combined 5-marker (*atpA*, *atpB*, *rbcL*, *trnL-F* and *rps4-trnS*) phylogenetic tree of Chinese *Adiantum* (in 132 accessions) comprised 6,497 nucleotides, of which 2,364 were variable (36.4%) and 1,772 were phylogenetically informative (27.3%). The MP analysis based on this dataset yielded one maximally parsimonious tree of 5,052 steps with a consistency index (CI) of 0.6033 and a retention index (RI) of 0.9198. The tree obtained from the BI analysis had a similar topology to the MP strict consensus tree (Fig 4). The monophyly of the series *Gravesiana* was also strongly supported here (100/1.0); however, all of its individuals were clustered into nine clades with a high support value (Fig 4). *A*. *gravesii* “*J*. *-M*. *Lu 451*”, *A*. *leveillei* “*J*. *-M*. *Lu 163*” and *A*. *lianxianense* “*J*. *-M*. *Lu 441*” in Lu *et al*. (2012) [2] were clustered in clade A with other individuals of *A*. *gravesii*. In addition, *A*. *mariesii* “*J*. *-M*. *Lu 120*” was clustered into clade D together with individuals of doubtful taxa “DT2.”. *A*. *chienii* “*J*. *-M*. *Lu 568*” was identical to “*FLG1-10*” and *A*. *juxtapositum* “*J*. *-M*. *Lu 120*.”

**Fig 5:**
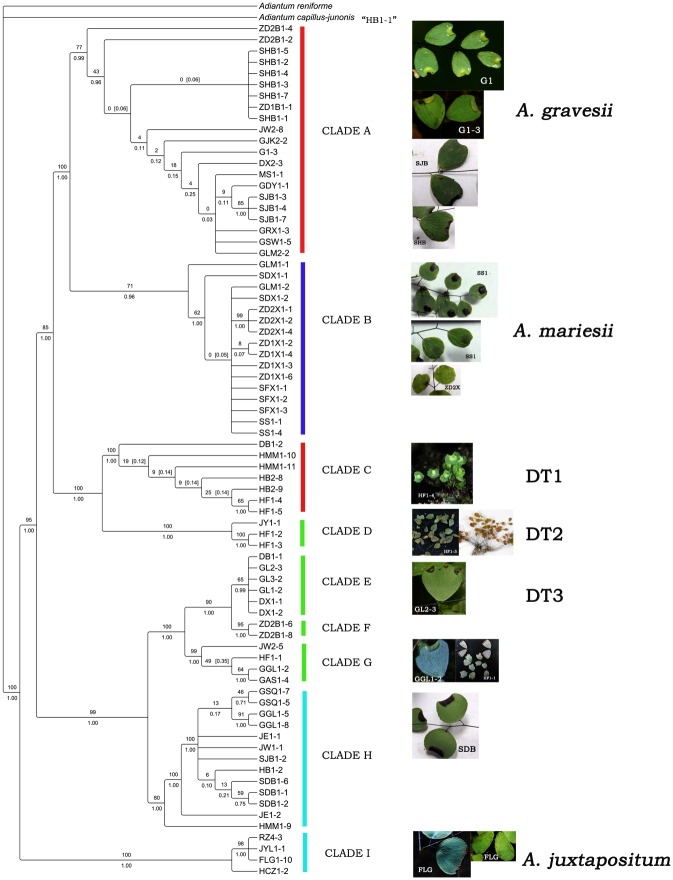
Strict consensus tree of six maximally parsimonious trees for series *Gravesiana* obtained from *atpA*, *atpB*, *rbcL*, *trnL-F*, *rps4-trnS* and *matK* sequences (tree length = 1,384 steps, CI = 0.8779, RI = 0. 0.9609). The bootstrap values were shown above the lines, and the Bayesian posterior probabilities were shown below the lines. Nine clades were labeled in columns and their own morphological characters were stated at right. For G1-3, GDY1-1, JW2-8, GJK2-2, …., front alphabets are the short names of different populations of taxa and the latter numbers represent single individuals as shown in S1 Table.

The trees of the series *Gravesiana* constructed with the combined 6-markers (*atpA*, *atpB*, *rbcL*, *trnL-F*, *trnS* and *matK*) included 78 taxa and were 7,140 nucleotides in length, of which 1,137 were variable (15.9%) and 490 were phylogenetically informative (6.9%). 1,384 steps were run to generate a maximally parsimonious tree; the consistency index (CI) was 0.8779, and the retention index (RI) was 0.9609. Nine main clades were identified in Fig 5, which was similar to the topology of the combined 5-marker tree but with high supported value. Twenty-one individuals of *A*. *gravesii* from twelve different populations were clustered into clade A, and 16 individuals of *A*. *mariesii* from 7 different populations were clustered into clade B. Seven individuals of the doubtful taxon “DT1” from four populations formed a clade (clade C). Three samples of “DT2” from two populations formed a new clade (clade D), and six individuals of “DT3” from three populations were clustered into clade E. All individuals of *A*. *juxtapositum* from four populations were clustered together (clade I).

### Morphological characters analyses

**Fig 6:**
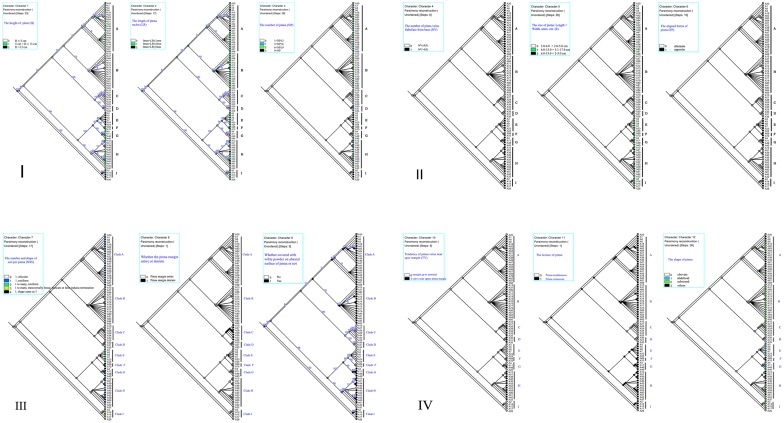
Twelve character optimisation over the 6-combined markers phylogeny tree (*atpA*, *atpB*, *rbcL*, *trnL-F*, *rps4-trnS* and *matK*), which were inferred by the Mesquite v. 2.71, using maximum parsimony.

In the 13 characters, scales among all individuals were similar; thus, we abandoned this character. The mapping to the phylogeny tree of the twelve other characters is visualized in Fig 6, and all characters of each clade are concluded in Table 2 to test whether the morphological characters of the species were consistent with the gene trees. It was clear that characters such as the height of the plant (H), pinna aligned forms, number of pinna (NP), pinna shape (SP), pinna margin (M), number of veins flabellate at base (NV), sori number and shape per pinna (NSS), pinna texture (T), and powder-covered or not on the abaxial surface of the pinna (P) are stable and reliable and can be distinguished through Table 2 and Fig 6. The size of the pinna was much relevant to its shape and the height of plants. Twenty-one individuals from thirteen different populations were clustered into clade A, and they shared some common traits: height usually longer than 5 cm, pinna alternate or opposite rarely, vein numbers flabellate at base greater than 4(5), sori 1, reniform or transversally linear, and with a notch at false indusia termination (Figs 5 and 6).

**Table 2:** Morphological characters of each clade concluded from the measured data.

	H (cm)	LR(mm)	FP	NP	S	SP	M	NV	TV	NSS	T	P
Clade A	middle, [0,13]	long, [0,5]	mostly alternate	3 to many	2.0–6.0×2.0–5.0; 6–15.0×5.0–17.0; 6–15.0×2.0–5.0;	subround or obdeltoid or oblong	entire or tiny wavy	>4(5)	curve near upper margin	1, reniform, rarely orbicular or transversally linear	coriaceous	yes
Clade B	short, [0, 5]	middle, [0, 3]	mostly alternate	3–9	small, 2.0–6.0 × 2.0–5.0	subround	entire	≤4(5)	curve near upper margin	1, orbicular, rarely reniform	coriaceous	yes
Clade C	short, [0, 5]	short, [0, 1]	alternate	1–3	small, 2.0–6.0 × 2.0–5.0	subround or obdeltoid	dentate	≤4(5)	curve near upper margin	1, orbicular, transversally linear, vertically linear	membranous	no
Clade D	Short, [0, 5]	short, [0, 1]	alternate	3–6	small, 2.0–6.0 × 2.0–5.0	obovate	entire or tiny wavy	≤4(5)	curve near upper margin	1, small, obicular	membranous	no
Clade E	tall, >13	middle, [0, 3]	opposite	6–9 or >9	large, 6–15.0 × 5.0–17.0	obdeltoid or fan-shaped	entire	>4(5)	straight up to terminal	1-many, reniform	coriaceous	no
Clade F	middle, [0,13]	middle, [0, 3]	opposite	6–9	large, 6–15.0 × 5.0–17.0	obdeltoid or fan-shaped	entire or tiny wavy	>4(5)	both	1, transversally linear, truncate at false indusial termination	coriaceous	no
Clade G	middle,[0, 13]	middle, [0, 3]	opposite	1–6	large, 6–15.0 × 5.01–17.0	obdeltoid	entire	>4(5)	both	1, transversally linear,	coriaceous	yes
Clade H	tall, >13	long, [0, 5]	mostly alternate	1–9	2.0–6.0×2.0–5.0; 6–15.0×5.0–17.0; 6–15.0×2.0–5.0;	subround or obdeltoid or oblong	entire or tiny wavy	>4(5)	curve near upper margin	1, reniform, rarely orbicular or transversally linear	coriaceous	yes
Clade I	tall, >13	middle, [0, 3]	opposite	6–9 or >9	large, 6–15.0 × 5.01–17.0	obdeltoid or fan-shaped	entire	>4(5)	straight up to terminal	1-many, transversally linear, truncate at false indusial termination	coriaceous	yes

H: Height; LR: Length of pinna stalk; FP: Pinna aligned forms; NP: Number of pinna; S: Pinna size; SP: Pinna shape; M: Pinna margin; NV: Number of veins flabellate at base; TV: Vein tendency near upper margin; NSS: Sori number and shape per pinna; T: Pinna texture; P: Powder-covered or not on the abaxial surface of the pinna.

Clade B shares common characters: all individuals were no more than 5 cm, with pinna alternate and sub-round and NV were less than 4(5), sori 1 for each pinna, and orbicular. All individuals in clade C can be easily identified by their much shorter height, NV less than 4 and dentate pinna margin. Individuals in clade D were featured by their membranous pinna texture and obovate pinna shape. For clade E, as shown in Figs 5 and 6, all samples included were large with pinna obdeltoid or flabellate, sori 1 to many (often 2–4), reniform, and NV 6–9. All samples of clade I were clustered together for their shared opposite pinna and 1-to-many sori per pinna and transversally linear indusia, truncate at false indusia termination. We did not analyze clade F considering its insufficient samples.

### SEM observation results

**Fig 7:**
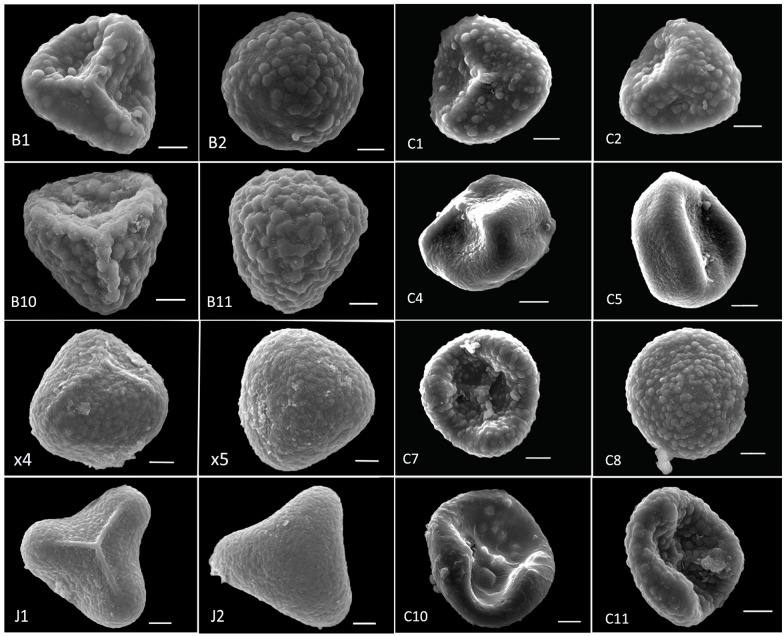
SEM observation of series *Gravesiana*. Clade A: B1, B2, B10 and B11; Clade B: x4, x5; Clade C: c10, c11; Clade H: c1-c8; Clade I: J1, J2.

The spore shapes of all taxa in series *Gravesiana* are similar in polar and equatorial views, but their surface ornamentations and spore sizes are clearly different. All spores are actinomorphic and trilete with polar surface triangle, and the equatorial surface is semicircular or super-semicircular. There are three kinds of surface ornamentations in all: psilate, rugate and verrucate (Fig 7). All spores of clade A are verrucate, while that of clade I are rugate, and that of the population “*HMM1*" in clade H are psilate. Interestingly, spore ornamentations of clade B are unapparent verrucate. Spores of clade C are rare and abortive. Although clade E share same spore ornamentations with clade A, sizes of its spores are bigger than that of clade A. Mean spore sizes of different populations in clade E and clade I are the biggest, whose length of equatorial axes varies from 52.0±5.0 μm to 61.87±2.7 μm and length of polar axes varies from 49.5 ±2.9 μm to 57.7 ±2.3 μm. Mean spore sizes of clade B are the second biggest, which are bigger than mean sizes of clade A. All spore sizes were presented in Table 3.

**Table 3:** Mean spores size of 15 natural populations in series *Gravesiana*, including length of polar axis and equatorial axis.

Population ID	Postion in the phylogenetic tree	Taxa	Collection sites	Length of equatorial axis (μm)	Length of polar axis (μm)
HCZ1	Clade I	*A*. *juxtapositum*	Chengjiang-kou in Zixin County, Chenzhou City, Hunan Province, China	61.87±2.7	57.7±2.27
FLG1	Clade I	*A*. *juxtapositum*	Guanzai Mountain, Liancheng County, Fujian Province, China	58.13±3.8	50.18±2.6
FLD1		*A*. *juxtapositum*	Dahongpao to Liuxiangjian in Wuyi Mountain Scenery spot, Fujian Province, China	55.98±4.13	47.53±2.30
RZ4	Clade I	*A*. *juxtapositum*	Danxia Mountain, Renhua County, Guangdong Province, China	52.0±5.0	49.5±2.9
DB1	Clade E	DT3	Jing-yang-cun primary school, Baoxu Town, Daxin county, Guangxi Province, China	58.83±2.5	50.32±5.29
ZD1X1	Clade B	*A*. *mariesii*	The trestle road along cliff in the Grand Canyon of Zhangjiajie Scenery Spot in Hunan Province, China	52.8±2.7	47.9±1.5
SS1	Clade B	*A*. *mariesii*	The cave in Shuitianba in Bamaoxi Township, Sangzhi County, Zhangjiajie City, Hunan Province, China	55.4±3.5	51.3±1.6
ZD1B1	Clade A	*A*. *gravesii*	The trestle road along cliff in the Grand Canyon of Zhangjiajie Scenery Spot in Hunan Province, China	54.73±4.27	50.08±2.1
MS1	Clade A	*A*. *gravesii*	Drippy Cave near the road in Shuijin Village, Mashan County, Guangxi Province, China	53.1±2.79	41.81±1.68
GLM2	Clade A	*A*. *gravesii*	Huangyanggou to Latan waterfall in Maolan Nature Reserve, Libo County, Guizhou Province, China	51.73±3.36	45.05±3.12
JW2-8	Clade A	*A*. *gravesii*	Genggang-tun in Xunma Village, Wuping Township, Jinxi County, Guangxi Province, China	48.8±2.38	40.33±2.65
GSW1	Clade A	*A*. *gravesii*	Air-raid shelter in Furong Mountain, Xihe Town, Wujiang District, Shaoguan City, Guangdong Province, China	47.57±3.33	39.49±2.16
DX2	Clade A	*A*. *gravesii*	Moist limestones under dense jungles of Xinfeng Village, Xialei Town, Daxin County, Guangxi Province, China	42.2±3.0	39.8±3.9
GSQ1	Clade H	?	Qingshui Bridge, Panlong Village, Shuicheng County, Guizhou Province, China	47.6±3.6	42±4.4
GGL1	Clade G, H	?	Leidayan, Sanjiang Farm, Guiyang City, Guizhou Province, China	44.3±3.45	39.81±2.9

Based on above molecular, morphological and spore analyses, clade A was considered to be *A*. *gravesii*, clade B was *A*. *mariesii* and clade H was *A*. *juxtapositum* without any doubt compared with their own type specimens (Figs 1 and 2). Clade C, D and E were treated as three new species, and we named them *Adiantum dentatum* A. H. Wang, F. G. Wang & F. W. Xing, *Adiantum obovatum* A. H. Wang, F. G. Wang & F. W. Xing and *Adiantum longzhouensis* A. H. Wang, F. G. Wang & F. W. Xing, respectively. For the three new species, the taxonomy, type, habitat and distribution, additional specimens examined (Paratypes), and IUCN conservation assessments are stated below.

### Taxonomy

**1. *Adiantum dentatum*** A. H. Wang, F. G. Wang & F. W. Xing **sp. nov.**

urn:lsid:ipni.org:names:77160197–1

**Fig 8:**
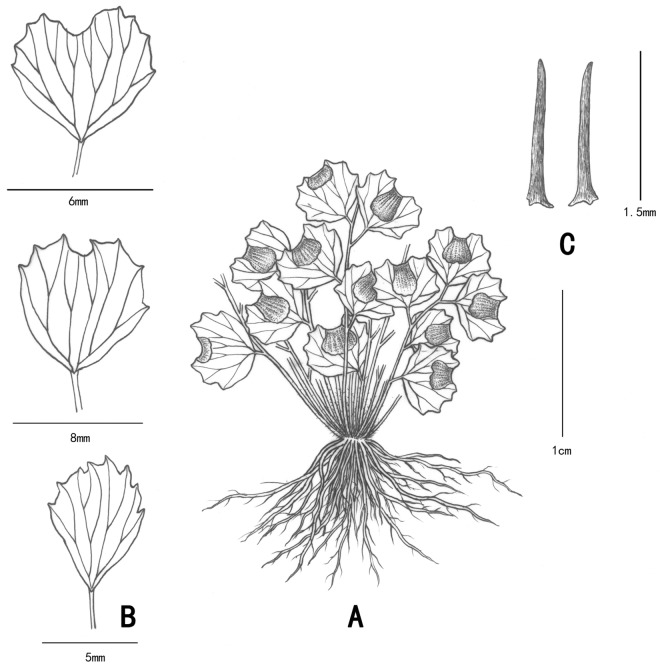
Morphological characters of *A*. *dentatum*. A: the whole plant; B: Pinna shapes; C: scales.

**Type:** China. Yang-zi-dong, Fengshan County, Guangxi. 24°23′21″N, 107°03′58"E, 13 June 2014, *WAH011* (holotypus, IBSC!). Fig 8

***A*. *dentatum*** differs from others for its fewer pinnae, obdeltoid or subovate pinna with prominent dentate margin from the broadest middle to apex and its variable orbicular or transversally linear sori.

Plants epilithic, small, 1–3 cm tall. Rhizomes short, erect, clothed with linear-lanceolate and black-brown scales, scales margin entire. Stipes densely tufted, 10–30 together, 1.5-9(15) mm long, ca. 0.5 mm thick, dark castaneous, as slender as silk. Lamina 1-pinnate, odd-pinnate or paripinnate, 1.5–2.5 cm long and ca. 2 cm broad; pinnae 1–3 pairs, 3–5, often 1–3, alternate, 2–6 mm apart, obliquely spreading; pinna ca. 2–8 mm long and broad, oval, subovate, obovate, broadly obovate or obdeltoid, cuneate or rounded-cuneate at base, margin entire on both lateral sides but dentate from the broadest middle to apex, upper margin with 1 shallow sinus in middle, texture subcoriaceous; stalk 1–2 mm, articulate, persistent after pinna fall; veins 4–6, flabellate at base, each again 2–4 forked and reached the tooth margin, visible on both surfaces. Sori 1 per pinna, orbicular or transversally linear, false indusia brown, upper margins flat and straight, slightly depressed, persistent, lower margins entire, sometimes with 1 distinct sinus in middle.

#### Habitat and distribution

The plants grow in moist chalk soil covered by black lichen or moss near the entrance to the karst cave Yang-zi-dong in Fengshan county, where drips exist. It has been found in karst caves in Huanjiang, Fengshan, Bama and Daxin in Guangxi Province.

#### Additional specimens examined (paratypes)

China. Guangxi: Ganhong Dong, Daluo Village, Nashe Township, Bama County, 13 June 2014, *WAH013*(IBSC); Jing-yang-cun primary school, Baoxu Town, Daxin county, 10 June 2014, *WAH012*(IBSC); Ming-li-tun, Mulun National Nature Reserve, Huanjiang County, 15 June 2014, *WAH014*(IBSC).

#### IUCN Conservation assessment

Although it grows well in the type locality, because of its restricted distribution region, small population size and low number of individuals in very small or restricted populations, ***A*. *dentatum*** should be considered endangered in accordance with the IUCN Red List criteria [28].

**2. *Adiantum obovatum*** A. H. Wang, F. G. Wang & F. W. Xing **sp. nov.**

urn:lsid:ipni.org:names:77160198–1

**Fig 9:**
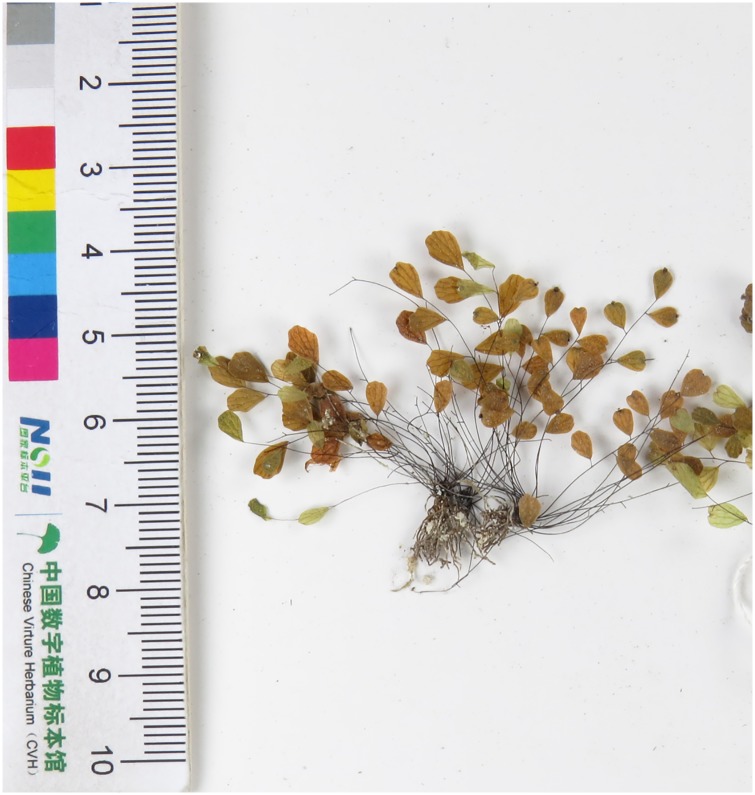
Morphological characters of *A*. *obovatum*.

**Type:** China. Yang-zi-dong, Fengshan County, Guangxi. 24°23′21″N, 107°03′58″E, 13 June 2014, *WAH016* (holotypus, IBSC!). Fig 9

This species can be easily distinguished from others for its membranous texture, petiole slender as hair, obovate pinna and 2–4 veins flabellate at base, and very small orbicular sori.

Plants epilithic, approximately 3.0–4.0 cm tall. Rhizomes short, erect, covered with linear-lanceolate and black-brown scales, scales margin entire. Stipes densely tufted, slender as hair, 0.8–1.8 cm long, ca. 0.5 mm thick, dark castaneous. Lamina 1-pinnate, odd-pinnate or paripinnate, 1.2–3.3 cm long and ca. 0.5–1.0 cm broad; pinnae 3–5, rarely 3, alternate; pinna ca. 2.5–6.0 × 2.0–4.0 mm, obovate, membranous, margin entire, upper margin with 1 shallow sinus in middle; stalk 0.5–1 mm, persistent after pinna fall; veins 2–4, flabellate at base, each again 2 forked and visible on both surfaces. Sori 1, orbicular, small.

#### Habitat and distribution

The plants grow in chalk soil covered by moss near the entrance to the karst cave Yang-zi-dong in Fengshan county. It has also been found to exist in the cave in Si-ming-xia, Yuexu town, Jinxi county.

#### Additional specimens examined (paratypes)

China. Guangxi: Si-ming-xia, Yuexu Town, Jinxi County, 11 June 2014, *WAH015*(IBSC).

#### IUCN Conservation assessment

Because of its restricted distribution region, small population size and low number of individuals in very small or restricted populations, ***A*. *obovatum*** should be considered endangered in accordance with the IUCN Red List criteria [28].

**3. *Adiantum longzhouensis*** A. H. Wang, F. G. Wang & F. W. Xing **sp. nov.**

urn:lsid:ipni.org:names:77160199–1

**Fig 10:**
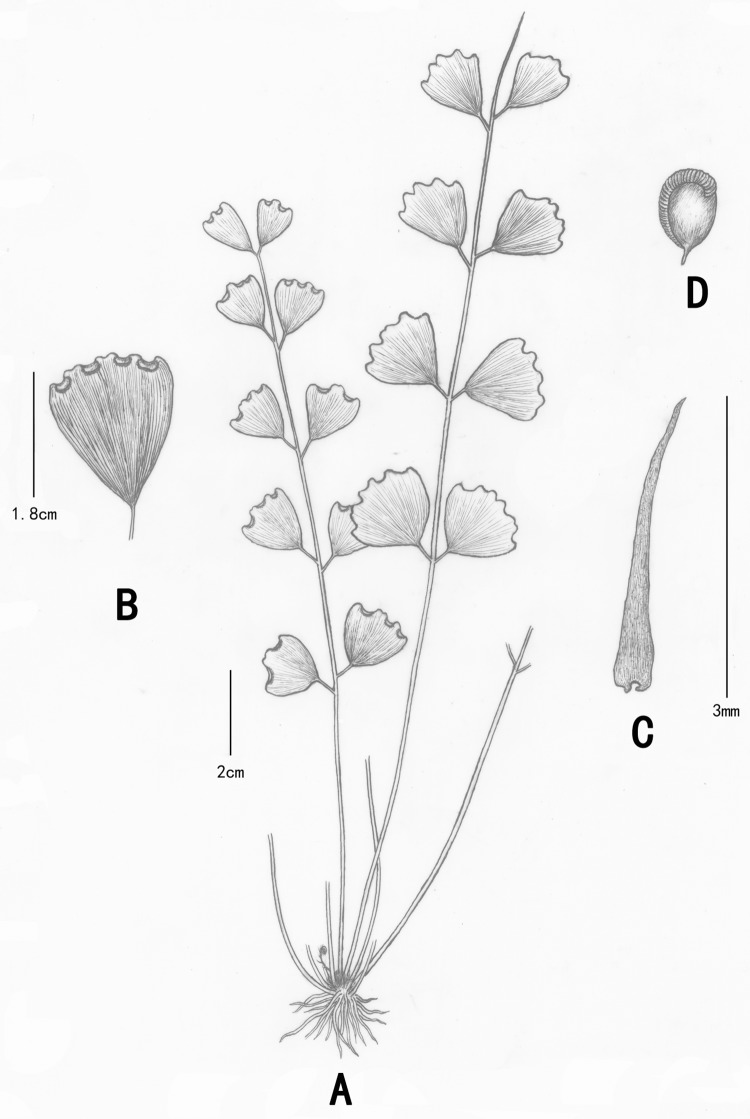
Morphological characters of *A*. *longzhouensis*. A: the whole plant; B: Pinna shapes and soris; C: scale; D: sporangium.

**Type:** China. Jing-yang-cun primary school, Baoxu Town, Daxin county, Guangxi 22°39′12″N, 106°57′27″E, 10 June 2014, *WAH017* (holotypus, IBSC!). Fig 10

The obdeltoid pinna and 1-many kidney-shaped sori per pinna of this species distinguish it from *A*. *juxtapositum and A*. *gravesii*.

Plants epilithic, 26.5 cm tall. Rhizomes short, erect, covered with lanceolate and black-brown scales, scales margin entire. Stipes tufted, 5–10 together, hard and stong, 5.6–11 cm long, ca. 1.0 mm thick, dark castaneous. Lamina 1-pinnate, odd-pinnate or paripinnate, 9–16 cm long and ca. 3.0–4.0 cm broad; pinnae 9–14, opposite, rarely alternate; ca. 10–18 × 10–21 mm, obdeltoid, coriaceous, margin entire, upper margin with 2–4 shallow sinus; stalk 0.5–1 mm, persistent after pinna fall; veins 7–10, flabellate at base, each again 2–4 forked, visible on both surfaces. Sori 2–4 per pinna, reniform.

#### Habitat and distribution

It grows in moist chalk soil on cliff under-forest in Longzhou county and Daxin county in Guangxi, China.

#### Additional specimens examined (paratypes)

China. Guangxi: Jing-yang-cun primary school, Baoxu Town, Daxin county, 10 June 2014, *WAH018* and *WAH019* (IBSC!); cliffs near the fifth single-log bridge in Longmeng, Sancunshan in the Nonggang Natural Reserve, Longzhou County, 9 June 2014, *WAH020*(IBSC); Sancunshan in the Nonggang Natural Reserve, Longzhou County, 9 June 2014, *WAH021*(IBSC); Cliffs near the second single-log bridge in Longmeng, Sancunshan in the Nonggang Natural Reserve, Longzhou County, 9 June 2014, *WAH022*(IBSC).

#### IUCN Conservation assessment

Its restricted distribution region, small population size and low number of individuals in very small or restricted populations make *A*. *longzhouensis* the endangered scale in accordance with the IUCN Red List criteria [28].

## Discussion

**Fig 11:**
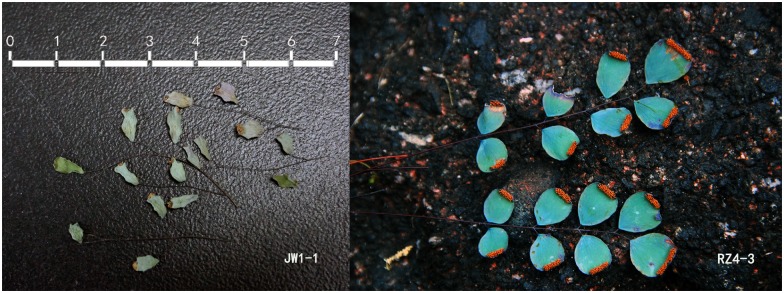
Morphological characters of “*JHW1-1*” and “*RZ4-3*” in clade H and clade I.

*A*. *obovatum* can be easily misidentified as *A*. *lianxianense* based on the description in Lin (1980) if we do not observe the type specimen of *A*. *lianxianense* carefully (Fig 1). *A*. *lianxianense* is coriaceous, and its sori are kidney-shaped rather than orbicular (Fig 1). Its similar individual “*JW1-1*” (Fig 11) clustered in clade H suggests that *A*. *lianxianense* is perhaps a variant of *A*. *gravesii*, which cannot be verified until we obtain DNA evidence of the type specimen. Samples of *A*. *chienii* have not been collected in the type locality, but similar ones were found in the Renhua Danxia mountain such as “*RZ4-3*”(Fig 11), which was clustered in *A*. *juxtapositum* (clade I). So it is possible that *A*. *chienii* and *A*. *juxtapositum* is of the same species. For clade G in Fig 5, the most remarkable difference from *A*. *longzhouensis* is that individuals in clade G are covered with white powder on the abaxial surface of the pinna while *A*. *longzhouensis* is not. However, we did not treat it as a new species considering its mixed characters: soris reniform or transversally linear with a truncate terminal at false indusium.

**Fig 12:**
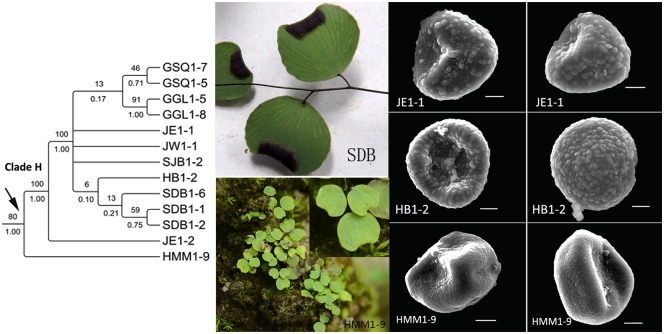
Morphological characters and spores ornamentations of the individuals (from different populations) in the clade H.

Height of the plant (H), pinna shape (SP) and number of veins flabellate at base (NV) of the four individuals “*JE1-1*” “*JE1-2*” “*HB1-2*” “*HMM1-9*” in clade H were same as *A*. *mariesii*, while their NSS (sori number and shape per pinna) were much more similar than *A*. *gravesii* (see Fig 12). Besides, morphological characters of plants in population *SDB* were similar to *A*. *juxtapositum* but their sori shapes were mixed characters: reniform or transversally linear with a truncate terminal at false indusium. Spores of “*JE1-1*” “*JE1-2*” “*HB1-2*” “*HMM1-9*” were scarce and seemed abortive, suggesting that gene exchange and hybridization may exist among *A*. *gravesii*, *A*. *juxtapositum* and *A*. *mariesii* considering the above morphological characters and spores results of clade G and H. It is interesting that three species exist in different microhabitats of the same cave in Yangzidong, Fengshan county (“*HF1-1*”, “*HF1-3*” and “*HF1-4*” in Fig 5). “*HF1-1*” grows in the chalk soil near the entrance to the cave, where drips are falling down. “*HF1-3*” lives in the cave without adequate sunshine and water. “*HF1-4*” exists in the young limestone within the cave accompanied by many moist green mosses. Then, what has caused the differentiation of these three sympatric species? We did not find any spores of “*HF1-2*” and “*HF1-3*”, spores of DT1 were also rare and abortive, so the doubtful species DT1 and DT2 are possibly hybrids. Or it is the result of species adapting to the soil, water, or sunshine or the interaction of these factors. At any rate, this interesting phenomenon has provided a good condition for us to study the mechanism of speciation in “terrestrial islands” later.

## Conclusions

There are six confirmed species in the series *Gravesiana* based on comprehensive analyses results of phylogeny and morphology. The species are *A*. *gravesii* (clade A), *A*. *mariesii* (clade B), *A*. *dentatum* (clade C, new species), *A*. *obovatum* (clade D, new species), *A*. *longzhouensis* (clade E, new species), and *A*. *juxtapositum* (clade I). Of the 13 characters, the height of the plant (H), pinna aligned forms (FP), number of pinna (NP), pinna margin (M), number of veins flabellate at base (NV), sori number and shape per pinna (NSS), pinna texture (T), and powder-covered or not on abaxial surface of pinna (P) are reliable, stable, and discrete characters for us to easily distinguish the six species above. Their retrieval keys are shown below. In addition, *A*. *leveillei* “*J*. *-M*. *Lu 163*” and *A*. *lianxianense* “*J*. *-M*. *Lu 441*” in Lu *et al*. (2012) was actually *A*. *gravesii*, and *A*. *mariesii* “*J*. *-M*. *Lu 120*” was *A*. *obovatum*. *A*. *chienii* “*J*. *-M*. *Lu 568*” was completely identical to *A*. *juxtapositum*.

1. plant shorter than 5 cm and number of veins flabellate at base less than 4(5)

 2. number of pinna less than or equal to 3, pinna margin dentate··························*A*. *dentatum*

 2. number of pinna more than 3, pinna margin entire

  3. pinna coriaceous, white powder-covered on abaxial surface, sori 1, large·············*A*. *mariesii*

  3. pinna membranous, without powder on abaxial surface, sori 1, very small··········*A*. *obovatum*

1. plant often taller than 5 cm and number of veins flabellate at base more than 4(5)

 4. pinna alternate, sori 1, reniform or transversally linear with sinus at indusium terminal·······························································································*A*. *gravesii*

 4. pinna opposite, rarely alternate, sori 1 to many, reniform or transversally linear

  5. pinna without white powder on abaxial surface, sori reniform····················*A*. *longzhouensis*

  5. pinna with white powder on abaxial surface, sori transversally linear, truncate at false indusium terminal······················································································*A*. *juxtapositum*

## Supporting information

S1 Table Voucher information and GenBank accession numbers for taxa used in the phylogenetic study on series *Gravesiana*. Index_ID, sequence_ID, voucher specimen (herbarium), collection locality, longitude and latitude, altitude and GenBank accession number in the order of *atpA*, *atpB*, *rbcL*, *trnL-F*, *rps4-trnS* and *matK*.
(DOCX)

S2 Table Voucher information and GenBank accession numbers for other taxa used in the phylogenetic study on *Adiantum*. Taxaname, sequence_ID, voucher specimen (herbarium), collection locality, and GenBank accession number in the order of *rbcL*, *atpB*, *atpA*, *trnL-F*, and *rps4-trnS*.
(DOCX)

S3 Table Original measured values of the morphological characters of series *Gravesiana*. H: Height; LR: Length of pinna stalk; FP: Pinna aligned forms; NP: Number of pinna; S: Pinna size; SP: Pinna shape; M: Pinna margin; NV: Number of veins flabellate at base; TV: Vein tendency near upper margin; NSS: Sori number and shape per pinna; T: Pinna texture; P: Powder-covered or not on the abaxial surface of the pinna.
(DOC)
