# Supplementary material for: Revision of series Gravesiana (Adiantum L.) based on morphological characteristics, spores and phylogenetic analyses
Source: PLoS One. 2017 Apr 5;12(4):e0172729. doi: 10.1371/journal.pone.0172729 (PMC5381765; doi:10.1371/journal.pone.0172729)
Supplement: S1 Table — Index_ID, sequence_ID, voucher specimen (herbarium), collection locality, longitude and latitude, altitude and GenBank accession number in the order of atpA, atpB, rbcL, trnL-F, rps4-trnS and matK. (DOCX) [file pone.0172729.s001.docx]

**Voucher information and GenBank accession numbers for taxa used in the phylogenetic study on series *Gravesiana*.** Index_ID, sequence_ID, voucher specimen (herbarium), collection locality, longitude and latitude, altitude and GenBank accession number in the order of *atpA*, *atpB*, *rbcL*, *trnL-F*, *rps4-trnS* and *matK*.

| **Index_ID** | **Sequence_ID** | **Collection Locality** | **Longitude and Latitude** | **Altitude (m)** | **Specimen_voucher** | **Genebank** | | | | | |
| --- | --- | --- | --- | --- | --- | --- | --- | --- | --- | --- | --- |
|  |  |  |  |  |  | **atpA** | **atpB** | **rbcL** | **trnL-F** | **rps4-trnS** | **matK** |
| A1 | G1-3 | Moist cliff behind the Qixing Park of Guilin City, Guangxi Province, China | 25°16′33″N,  110°18′51″E |  | WAH054 | KY382659 | KY429033 | KY450804 | KY450922 | KY451008 | KY461645 |
| A2 | RZ4-3 | On the cliffs from Yangyuanshi to Tongtaiqiao in Danxia Mountain, Renhua County, Guangdong Province, China | 25°02′49″N, 113°44′51″E | 160 | WAH029 | KY382660 | KY429034 | KY450805 | KY450886 | KY450972 | KY461646 |
| A3 | DB1-1 | Jing-yang-cun primary school, Baoxu Town, Daxin county, Guangxi Province, China | 22°39′12″N, 106°57′27″E | 230 | WAH019 | KY382661 | KY429035 | KY450806 | KY450906 | KY451006 | KY461650 |
| A4 | HF1-1 | Yangzi Dong, Fengshan County, Guangxi Province, China | 24°23′21″N, 107°03′58″E | 594 | WAH055 | KY382662 | KY429036 | KY450807 | KY450907 | KY450969 | KY461651 |
| A5 | GL2-3 | Sancunshan in Nonggang Natural Reserve, Longzhou County, Guangxi Province, China | 22°29′11″N,106°56′03″E | \| 277 \| 20140609 \| \| --- \| --- \| | WAH021 | KY382663 | KY450799 | KY450808 | KY450908 | KY450968 | KY461652 |
| A6 | GL3-2 | Cliffs near the fifth single-log bridge in Longmeng, Sancunshan in Nonggang Natural Reserve, Longzhou County, Guangxi Province, China | 22°29′12″N,  106°56′06″E | 249 | WAH020 | KY382664 | KY429037 | KY450809 | KY450913 | KY450966 | KY461653 |
| A7 | GL1-2 | Cliffs near the second single-log bridge in Longmeng, Sancunshan in Nonggang Natural Reserve, Longzhou County, Guangxi Province, China | 22°29′13″N,  106°56′08″E | 187 | WAH022 | KY382665 | KY429038 | KY450810 | KY450914 | KY450993 | KY461654 |
| A8 | DX2-3 | Moist limestones under dense jungles of Xinfeng Village, Xialei Town, Daxin County, Guangxi Province, China | 22°53′52″N, 106°43′05″E | 557 | WAH034 | KY382666 | KY429039 | KY450811 | KY450919 | KY451007 | KY461655 |
| A9 | MS1-1 | Drippy Cave near the road in Shuijin Village, Mashan County, Guangxi Province, China | 23°35′24″N, 108°19′06″E | 232 | WAH037 | KY382667 | KY450795 | KY450812 | KY450920 | KY451009 | KY461656 |
| A10 | HB2-8 | Ganhong Dong, Daluo Village, Nashe Township, Bama County, Guangxi Province, China | 24°18′44″N, 106°59′55″E | 187 | WAH013 | KY382668 | KY450794 | KY450801 | KY450878 | KY450955 | KY461657 |
| A11 | GGL1-2 | Leidayan, Sanjiang Farm, Guiyang City, Guizhou Province, China | 26°41′49″N, 106°49′31″E |  | WAH041 | KY382669 | KY429040 | KY450813 | KY450917 | KY450965 | KY461658 |
| A12 | GSQ1-7 | Qingshui Bridge, Panlong Village, Shuicheng County, Guizhou Province, China | 26°18′04″N, 105°07′08″E | 1056 | WAH031 | KY382670 | KY450798 | KY450814 | KY450892 | KY450983 | KY461659 |
| A13 | GLM1-1 | Banyan Cave, Maolan Nature Reserve, Libo County, Guizhou Province, China | 25°21′50″N, 107°56′10″E | 763 | WAH038 | KY382671 | KY450797 | KY450815 | KY450905 | KY451018 |  |
| A14 | JYL1-1 | Zhangjia Dong outside of the Dragon and Tiger Mountain, Yingtan City, Jiangxi Province, China | 28°06′35″N,  116°59′12″E | 79 | WAH053 | KY382672 | KY429041 | KY450816 | KY450885 | KY450973 | KY461647 |
| A15 | FLG1-10 | Sandie Tan to Xianren Valley in Guanzai Mountain, Liancheng County, Fujian Province, China | 25°42′31″N,116°47′20″E | 396 | WAH030 | KY382673 | KY429042 | KY450802 | KY450887 | KY450974 | KY461648 |
| A17 | HCZ1-2 | Near the highway to Chengjiang-kou in Zixin County, Chenzhou City, Hunan Province, China | 26°01′38″N, 113°10′18″E |  | WAH028 | KY382674 | KY429043 | KY450803 | KY450888 | KY450981 | KY461649 |
| A18 | GDY1-1 | Hengshi-tang town, Yingde City, Guangdong Province, China |  |  | WAH056 | KY382675 | KY429044 | KY450817 | KY450921 | KY450995 |  |
| A21 | GRX1-3 | Xiyun temple, Daqiao Town, Ruyuan County, Guangdong Province, China | 24°53′48″N, 113°07′31″E | 599 | WAH057 | KY382676 | KY429045 | KY450818 | KY450923 | KY451017 | KY461660 |
| A22 | GSW1-5 | Air-raid shelter in Furong Mountain, Xihe Town, Wujiang District, Shaoguan City, Guangdong Province, China | 24°46′28″N, 113°33′18″E | 49 | WAH036 | KY382677 | KY429046 | KY450819 | KY450924 | KY450996 | KY461661 |
| A23 | DX1-1 | Around the Ming-shi-tian-yuan of the Yanbian Road in Daxin County, Guangxi Province, China | 22°43′01″N, 106°54′19″E | 238 | WAH017 | KY382678 | KY429047 | KY450820 | KY450909 | KY451005 | KY461662 |
| A24 | DX1-2 | Around the Ming-shi-tian-yuan of the Yanbian Road in Daxin County, Guangxi Province, China | 22°43′01″N, 106°54′19″E | 238 | WAH018 | KY382679 | KY450800 | KY450821 | KY450910 | KY451004 | KY461663 |
| A25 | DB1-2 | Jing-yang-cun primary school, Baoxu Town, Daxin county, Guangxi Province, China | 22°39′12″N, 106°57′27″E | 230 | WAH012 | KY382680 | KY429048 | KY450822 | KY450880 | KY450957 | KY461664 |
| A26 | HB1-1 | The cave near Ganhong Dong, Daluo Village, Nashe Township, Bama County, Guangxi Province, China | 24°18′35″N, 106°59′41″E | 481 | WAH023 | KY382681 | KY450793 | KY450823 | KY450953 |  | KY461665 |
| A27 | HB2-9 | Ganhong Dong, Daluo Village, Nashe Township, Bama County, Guangxi Province, China | 24°18′44″N, 106°59′55″E | 187 | WAH013 | KY382682 | KY429049 | KY450824 | KY450879 | KY450956 |  |
| A28 | HF1-4 | Yangzi Dong, Fengshan County, Guangxi Province, China | 24°23′21″N, 107°03′58″E | 594 | WAH011 | KY382683 | KY429050 | KY450825 | KY450881 | KY450958 |  |
| A29 | HF1-5 | Yangzi Dong, Fengshan County, Guangxi Province, China | 24°23′21″N, 107°03′58″E | 594 | WAH011 | KY382684 | KY429051 | KY450826 | KY450882 | KY450959 | KY461666 |
| A30 | HF1-2 | Yangzi Dong, Fengshan County, Guangxi Province, China | 24°23′21″N, 107°03′58″E | 605 | WAH016 | KY382685 | KY429052 | KY450827 | KY450901 | KY450975 | KY461642 |
| A31 | HF1-3 | Yangzi Dong, Fengshan County, Guangxi Province, China | 24°23′21″N, 107°03′58″E | 605 | WAH016 | KY382686 | KY429053 | KY450828 | KY450902 | KY450976 | KY461643 |
| A32 | HMM1-9 | Ming-li-tun, Mulun National Nature Reserve, Huanjiang County, Guangxi Province, China | 25°06′02″N, 107°58′09″E | 158 | WAH024 | KY382687 | KY429054 | KY450829 | KY450954 | KY450971 | KY461667 |
| A33 | HMM1-10 | Ming-li-tun, Mulun National Nature Reserve, Huanjiang County, Guangxi Province, China | 25°06′02″N, 107°58′09″E | 158 | WAH014 | KY382688 | KY429055 | KY450830 | KY450883 | KY450960 | KY461668 |
| A34 | HMM1-11 | Ming-li-tun, Mulun National Nature Reserve, Huanjiang County, Guangxi Province, China | 25°06′02″N, 107°58′09″E | 158 | WAH014 | KY382689 | KY429056 | KY450831 | KY450884 | KY450961 | KY461669 |
| A35 | JY1-1 | Si-ming-xia,Yuexu Town, Jingxi County, Guangxi, China |  |  | WAH015 | KY382690 | KY429057 | KY450832 | KY450903 | KY450977 | KY461644 |
| A36 | JE1-1 | The cave in E-quan Village, Jinxi County, Guangxi Province, China | 23°06′12″N,  106°22′48″E | 398 | WAH039 | KY382691 | KY429058 | KY450833 | KY450893 | KY450978 | KY461670 |
| A37 | GLM1-2 | Banyan Cave, Maolan Nature Reserve, Libo County, Guizhou Province, China | 25°21′50″N, 107°56′10″E | 763 | WAH038 | KY382692 | KY429059 | KY450834 | KY450904 | KY451019 | KY461671 |
| A38 | GLM2-2 | Huangyanggou to Latan waterfall in Maolan Nature Reserve, Libo County, Guizhou Province, China | 25°15′54″N, 108°03′48″E | 467 | WAH035 | KY382693 | KY429060 | KY450835 | KY450952 | KY450994 | KY461672 |
| A39 | GGL1-5 | Leidayan, Sanjiang Farm, Guiyang City, Guizhou Province, China | 26°41′49″N, 106°49′31″E |  | WAH042 | KY382694 | KY429061 | KY450836 | KY450890 | KY450985 | KY461673 |
| A40 | GGL1-8 | Leidayan, Sanjiang Farm, Guiyang City, Guizhou Province, China | 26°41′49″N, 106°49′31″E |  | WAH042 | KY382695 | KY429062 | KY450837 | KY450891 | KY450986 | KY461674 |
| A42 | JE1-2 | The cave in E-quan Village, Jinxi County, Guangxi Province, China | 23°06′12″N,  106°22′48″E | 398 | WAH039 | KY382696 | KY429063 | KY450838 | KY450894 | KY450979 | KY461675 |
| A43 | JW1-1 | Nongwei-tun in Nongguang Village, Wuping Township, Jinxi County, Guangxi Province, China | 23°11′29″N, 106°32′15″E | 259 | WAH025 | KY382697 | KY429064 | KY450839 | KY450895 | KY450980 | KY461676 |
| A45 | JW2-5 | Genggang-tun in Xunma Village, Wuping Township, Jinxi County, Guangxi Province, China | 23°11′46″N, 106°30′16″E | 738 | WAH027 | KY382698 | KY429065 | KY450840 | KY450911 | KY450970 | KY461677 |
| A47 | JW2-8 | Genggang-tun in Xunma Village, Wuping Township, Jinxi County, Guangxi Province, China | 23°11′46″N, 106°30′16″E | 738 | WAH026 | KY382699 | KY429066 | KY450841 | KY450925 | KY450997 | KY461678 |
| A48 | GAS1-4 | The road from Longgong to Xuantang in Anshuan City, Guizhou Province, China | 26°07′00″N, 105°52′46″E |  | WAH033 | KY382700 | KY429067 | KY450842 | KY450912 | KY450992 | KY461679 |
| A49 | GJK2-2 | Baishui cave, Minxiao river, Jiangkou County, Guizhou Province, China | 27°39′24″N, 108°47′28″E | 423 | WAH032 | KY382701 | KY429068 | KY450843 | KY450926 | KY451010 | KY461680 |
| A51 | GSQ1-5 | Qingshui Bridge, Panlong Village, Shuicheng County, Guizhou Province, China | 26°18′04″N, 105°07′08″E | 1056 | WAH031 | KY382702 | KY429069 | KY450844 | KY450889 | KY450984 | KY461681 |
| A52 | HB1-2 | The cave near Gan-hong Dong, Daluo Village, Nashe Township, Bama County, Guangxi Province, China | 24°18′35″N, 106°59′41″E | 481 | WAH040 | KY382703 | KY429070 | KY450845 | KY450896 | KY450982 | KY461682 |
| A53 | SDB1-6 | The big cave around the road beside the Jiashi River in Sangzhi County, Zhangjiajie City, Hunan Province, China | 29°24′22″N, 110°09′46″E | 421 | WAH046 | KY382704 | KY429071 | KY450846 | KY450897 | KY450987 | KY461683 |
| A54 | SHB1-5 | Huangliantai Village, Bamaoxi Township, Sangzhi County, Zhangjiajie City, Hunan Province, China | 29°24′10″N, 110°09′57″E |  | WAH047 | KY382705 | KY429072 | KY450847 | KY450927 | KY451011 | KY461684 |
| A55 | SHB1-2 | Huangliantai Village, Bamaoxi Township, Sangzhi County, Zhangjiajie City, Hunan Province, China | 29°24′10″N, 110°09′57″E |  | WAH047 | KY382706 | KY429073 | KY450848 | KY450928 | KY451012 | KY461685 |
| A56 | SHB1-4 | Huangliantai Village, Bamaoxi Township, Sangzhi County, Zhangjiajie City, Hunan Province, China | 29°24′10″N, 110°09′57″E |  | WAH047 | KY382707 | KY450796 | KY450849 | KY450929 | KY451013 | KY461686 |
| A57 | SHB1-3 | Huangliantai Village, Bamaoxi Township, Sangzhi County, Zhangjiajie City, Hunan Province, China | 29°24′10″N, 110°09′57″E |  | WAH047 | KY382708 | KY429074 | KY450850 | KY450930 | KY451014 | KY461687 |
| A58 | SHB1-7 | Huangliantai Village, Bamaoxi Township, Sangzhi County, Zhangjiajie City, Hunan Province, China | 29°24′10″N, 110°09′57″E |  | WAH047 | KY382709 | KY429075 | KY450851 | KY450931 | KY451015 | KY461688 |
| A59 | SDB1-1 | The big cave around the road beside the Jiashi River in Sangzhi County, Zhangjiajie City, Hunan Province, China | 29°24′22″N, 110°09′46″E | 421 | WAH046 | KY382710 | KY429076 | KY450852 | KY450898 | KY450988 | KY461689 |
| A60 | SDB1-2 | The big cave around the road beside the Jiashi River in Sangzhi County, Zhangjiajie City, Hunan Province, China | 29°24′22″N, 110°09′46″E | 421 | WAH045 | KY382711 | KY429077 | KY450853 | KY450899 | KY450989 | KY461690 |
| A61 | SJB1-2 | Moist limestones near Jiashi River in Sangzhi County, Zhangjiajie City, Hunan Province, China | 29°28′42″N, 109°55′59″E | 351 | WAH051 | KY382712 | KY429078 | KY450854 | KY450900 | KY450990 | KY461691 |
| A62 | SJB1-3 | Moist limestones near Jiashi River in Sangzhi County, Zhangjiajie City, Hunan Province, China | 29°28′42″N, 109°55′59″E | 351 | WAH051 | KY382713 | KY429079 | KY450855 | KY450932 | KY450998 | KY461692 |
| A63 | SJB1-4 | Moist limestones near Jiashi River in Sangzhi County, Zhangjiajie City, Hunan Province, China | 29°28′42″N, 109°55′59″E | 351 | WAH051 | KY382714 | KY429080 | KY450856 | KY450933 | KY450999 | KY461693 |
| A64 | SJB1-7 | Moist limestones near Jiashi River in Sangzhi County, Zhangjiajie City, Hunan Province, China | 29°28′42″N, 109°55′59″E | 351 | WAH051 | KY382715 | KY429081 | KY450857 | KY450934 | KY451000 | KY461694 |
| A65 | SDX1-1 | The big cave around the road beside the Jiashi River in Sangzhi County, Zhangjiajie City, Hunan Province, China | 29°24′22″N, 110°09′46″E | 421 | WAH052 | KY382716 | KY429082 | KY450858 | KY450935 | KY451020 | KY461695 |
| A66 | SDX1-2 | The big cave around the road beside the Jiashi River in Sangzhi County, Zhangjiajie City, Hunan Province, China | 29°24′22″N, 110°09′46″E | 421 | WAH052 | KY382717 | KY429083 | KY450859 | KY450936 | KY451021 | KY461696 |
| A67 | ZD2B1-2 | The trestle road along cliff in the Grand Canyon of Zhangjiajie Scenery Spot in Hunan Province, China | 29°24′35″N, 110°41′40″E |  | WAH048 | KY382718 | KY429084 | KY450860 | KY450937 | KY451002 | KY461697 |
| A68 | ZD2B1-4 | The trestle road along cliff in the Grand Canyon of Zhangjiajie Scenery Spot in Hunan Province, China | 29°24′35″N, 110°41′40″E |  | WAH048 | KY382719 | KY429085 | KY450861 | KY450938 | KY451003 | KY461698 |
| A69 | ZD2B1-6 | The trestle road along cliff in the Grand Canyon of Zhangjiajie Scenery Spot in Hunan Province, China | 29°24′35″N, 110°41′40″E |  | WAH048 | KY382720 | KY429086 | KY450862 | KY450915 | KY450967 | KY461699 |
| A70 | ZD2B1-8 | The trestle road along cliff in the Grand Canyon of Zhangjiajie Scenery Spot in Hunan Province, China | 29°24′35″N, 110°41′40″E |  | WAH048 | KY382721 | KY429087 | KY450863 | KY450916 | KY450991 | KY461700 |
| A71 | ZD2X1-1 | The trestle road along cliff in the Grand Canyon of Zhangjiajie Scenery Spot in Hunan Province, China | 29°24′35″N, 110°41′40″E | 247 | WAH043 | KY382722 | KY429088 | KY450864 | KY450918 | KY450962 | KY461701 |
| A72 | ZD2X1-2 | The trestle road along cliff in the Grand Canyon of Zhangjiajie Scenery Spot in Hunan Province, China | 29°24′35″N, 110°41′40″E | 247 | WAH043 | KY382723 | KY429089 | KY450865 | KY450939 | KY450963 | KY461702 |
| A73 | ZD2X1-4 | The trestle road along cliff in the Grand Canyon of Zhangjiajie Scenery Spot in Hunan Province, China | 29°24′35″N, 110°41′40″E | 247 | WAH043 | KY382724 | KY429090 | KY450866 | KY450940 | KY450964 | KY461703 |
| A74 | ZD1X1-2 | The trestle road along cliff in the Grand Canyon of Zhangjiajie Scenery Spot in Hunan Province, China | 29°24′35″N, 110°41′40″E | 186 | WAH044 | KY382725 | KY429091 | KY450867 | KY450941 | KY451022 | KY461704 |
| A75 | ZD1X1-3 | The trestle road along cliff in the Grand Canyon of Zhangjiajie Scenery Spot in Hunan Province, China | 29°24′35″N, 110°41′40″E | 186 | WAH044 | KY382726 | KY429092 | KY450868 | KY450942 | KY451023 | KY461705 |
| A76 | ZD1X1-4 | The trestle road along cliff in the Grand Canyon of Zhangjiajie Scenery Spot in Hunan Province, China | 29°24′35″N, 110°41′40″E | 186 | WAH044 | KY382727 | KY429093 | KY450869 | KY450943 | KY451024 | KY461706 |
| A77 | ZD1X1-6 | The trestle road along cliff in the Grand Canyon of Zhangjiajie Scenery Spot in Hunan Province, China | 29°24′35″N, 110°41′40″E | 186 | WAH044 | KY382728 | KY429094 | KY450870 | KY450944 | KY451025 | KY461707 |
| A78 | ZD1B1-1 | The trestle road along cliff in the Grand Canyon of Zhangjiajie Scenery Spot in Hunan Province, China | 29°24′35″N, 110°41′40″E | 186 | WAH048 | KY382729 | KY429095 | KY450871 | KY450945 | KY451001 | KY461708 |
| A79 | SFX1-1 | Feilong cave, Lianjiawan Village, Wudaoshui Town, Sangzhi County, Zhangjiajie City, Hunan Province, China | 29°42′04″N, 110°04′04″E | 377 | WAH049 | KY382730 | KY429096 | KY450872 | KY450946 | KY451026 | KY461709 |
| A80 | SFX1-2 | Feilong cave, Lianjiawan Village, Wudaoshui Town, Sangzhi County, Zhangjiajie City, Hunan Province, China | 29°42′04″N, 110°04′04″E | 377 | WAH049 | KY382731 | KY429097 | KY450873 | KY450947 | KY451027 | KY461710 |
| A81 | SFX1-3 | Feilong cave, Lianjiawan Village, Wudaoshui Town, Sangzhi County, Zhangjiajie City, Hunan Province, China | 29°42′04″N, 110°04′04″E | 377 | WAH049 | KY382732 | KY429098 | KY450874 | KY450948 | KY451028 | KY461711 |
| A82 | SHB1-1 | Huangliantai Village, Bamaoxi Township, Sangzhi County, Zhangjiajie City, Hunan Province, China | 29°24′10″N, 110°09′57″E |  | WAH047 | KY382733 | KY429099 | KY450875 | KY450949 | KY451016 | KY461712 |
| A83 | SS1-1 | The cave in Shuitianba in Bamaoxi Township, Sangzhi County, Zhangjiajie City, Hunan Province, China | 29°39′14″N, 110°03′09″E | 323 | WAH050 | KY382734 | KY429100 | KY450876 | KY450950 | KY451029 | KY461713 |
| A84 | SS1-4 | The cave in Shuitianba in Bamaoxi Township, Sangzhi County, Zhangjiajie City, Hunan Province, China | 29°39′14″N, 110°03′09″E | 323 | WAH050 | KY382735 | KY429101 | KY450877 | KY450951 | KY451030 | KY461714 |
