# Supplementary material for: Revision of series Gravesiana (Adiantum L.) based on morphological characteristics, spores and phylogenetic analyses
Source: PLoS One. 2017 Apr 5;12(4):e0172729. doi: 10.1371/journal.pone.0172729 (PMC5381765; doi:10.1371/journal.pone.0172729)
Supplement: S2 Table — Taxaname, sequence_ID, voucher specimen (herbarium), collection locality, and GenBank accession number in the order of rbcL, atpB, atpA, trnL-F, and rps4-trnS. (DOCX) [file pone.0172729.s002.docx]

**Voucher information and GenBank accession numbers for taxa used in the phylogenetic study on *Adiantum*.** Taxaname, sequence_ID, voucher specimen (herbarium), collection locality, and GenBank accession number in the order of *rbcL*, *atpB*, *atpA*, *trnL-F*, and *rps4-trnS*.

| Taxaname | Sequence_ID | Specimen_voucher |  | GenBank accession number | | | | Collection Locality |
| --- | --- | --- | --- | --- | --- | --- | --- | --- |
|  |  |  | rbcL | atpB | atpA | trnL-F | rps4-trnS |  |
| *Adiantum aethiopicum* |  | J. Wen 10780 (US) | JF935350 | JF935432 | JF937305 | JF980695 | JF980616 | New Zealand |
| *Adiantum aleuticum* |  | Heutte s.n. (US) | JF935362 | JF935447 | JF937320 | JF980709 | JF980631 | Alaska, USA |
| *Adiantum bonatianum* |  | J.-M. Lu 216 (KUN) | JF935294 | JF935371 | JF937247 | JF980639 | JF980556 | Yunnan, China |
| *Adiantum capillus-junonis* |  | J.-M. Lu 111 (KUN) | JF935314 | JF935395 | JF937269 | JF980662 | JF980578 | Guangxi, China |
| *Adiantum capillus-veneris* |  | J. Wen 8192 (US) | JF935332 | JF935413 | JF937286 | JF980676 | JF980597 | Chongqing, China |
| *Adiantum caudatum* |  | J.-M. Lu 209 (KUN) | JF935296 | JF935373 | JF937249 | JF980641 | JF980558 | Hainan, China |
| *Adiantum caudatum* |  | J.-M. Lu 050 (KUN) | JF935297 | JF935374 | JF937250 | JF980642 | JF980559 | Yunnan, China |
| *Adiantum chilense* |  | J. Wen 7313 (US) | JF935336 | JF935418 | JF937291 | JF980681 | JF980602 | Nuble, Chile |
| *Adiantum cuneatum* |  | J. Wen 10119 (US) | JF935339 | JF935421 | JF937294 | JF980684 | JF980605 | West Java, Indonesia |
| *Adiantum davidii* |  | J.-M. Lu 344 (KUN) | JF935310 | JF935391 | JF937265 | JF980659 | JF980574 | Shanxi, China |
| *Adiantum davidii* var. *longispinum* |  | J.-M. Lu 247 (KUN) | JF935292 | JF935369 | JF937245 | JF980638 | JF980554 | Yunnan, China |
| *Adiantum diaphanum* |  | J. Wen 10727 (US) | JF935301 | JF935439 | JF937312 | JF980702 | JF980623 | Indonesia |
| *Adiantum edentulum* |  | J.-M. Lu 222 (KUN) | JF935291 | JF935368 | JF937244 | JF980637 | JF980553 | Yunnan, China |
| *Adiantum menglianense* |  | J.-M. Lu 114 (KUN) | JF935311 | JF935392 | JF937266 | JF980660 | JF980575 | Guangxi, China |
| *Adiantum excisum* |  | J. Wen 7326 (US) | JF935311 | JF935419 | JF937292 | JF980682 | JF980603 | Concepcion, chile |
| *Adiantum fengianum* |  | J.-M. Lu 228 (KUN) | JF935308 | JF935388 | JF937262 | JF980656 | JF980571 | Yunnan, China |
| *Adiantum fimbriatum* |  | J.-M. Lu 215 (KUN) | JF935321 | JF935402 | JF937275 |  | JF980585 | Yunnan, China |
| *Adiantum flabellulatum* |  | J. Wen 6585 (US) | JF935325 | JF935406 | JF937279 | JF980670 | JF980589 | Hainan, China |
| *Adiantum gravesii* |  | J.-M. Lu 451 (KUN) | JF935317 | JF935398 | JF937272 | JF980664 | JF980581 | Guangdong, China |
| *Adiantum hispidulum* |  | J. Wen 10771 (US) | JF935349 | JF935431 | JF937304 | JF980694 | JF980615 | Virginia cultivated, USA |
| *Adiantum induratum* | 6 | Hrr_001 (IBSC) | JF935309 | KJ742755 | KJ742798 | KJ779992 | KJ779995 | Hainan, China |
| *Adiantum leveillei* |  | J.-M. Lu 163 (KUN) | JF935313 | JF935394 | JF937268 | JF980661 | JF980577 | Guangxi, China |
| *Adiantum lianxianense* |  | J.-M. Lu 441 (KUN) | JF935306 | JF935385 | JF937259 | JF980653 | JF980569 | Guangdong, China |
| *Adiantum malesianum* | 10 | Hrr_002 (IBSC) | JF935297 | KJ742756 | KJ742799 | KJ779993 | KJ779994 | Guangzhou, Guangdong, China |
| *Adiantum mariesii* |  | J.-M. Lu 120 (KUN) | JF935302 | JF935380 | JF937255 | JF980648 | JF980564 | Guangxi, China |
| *Adiantum myriosorum* |  | J.-M. Lu 297 (KUN) | JF935359 | JF935444 | JF937317 | JF980706 | JF980628 | Chongqing, China |
| *Adiantum pedatum* |  | J.-M. Lu 343 (KUN) | JF935360 | JF935445 | JF937318 | JF980707 | JF980629 | Shanxi, China |
| *Adiantum philippense* |  | J. Wen 8257 (US) | JF935330 | JF935412 | JF937285 | JF980675 | JF980595 | Luzon, the Philippines |
| *Adiantum raddianum* |  | J. Wen 9521 (US) | JF935323 | JF935404 | JF937277 | JF980668 | JF980587 | Antsiranana, Madagascar |
| *Adiantum reniforme* | TB31 | Wah011 (IBSC) | KJ742772 | KJ742740 | KJ742783 | KJ779977 | KJ780005 | Barranco del infierno, Tenerife, Canary Islands, Spain |
| *Adiantum reniforme* | LPB1 | Wah012 (IBSC) | KJ742766 | KJ742741 | KJ742784 | KJ779985 | KJ780006 | Bermudez, La Palma, Canary Islands, Spain |
| *Adiantum reniforme* | LPB19 | Wah012 (IBSC) | KJ742765 | KJ742742 | KJ742785 | KJ779986 | KJ780007 | Bermudez, La Palma, Canary Islands, Spain |
| *Adiantum reniforme* | LPB27 | Wah012 (IBSC) | KJ742770 | KJ742743 | KJ742786 | KJ779987 | KJ780008 | Bermudez, La Palma, Canary Islands, Spain |
| *Adiantum reniforme* | LPC7 | Wah013 (IBSC) | KJ742769 | KJ742744 | KJ742787 | KJ779988 | KJ780009 | Cubo dela Galga, La Palma, Canary Islands, Spain |
| *Adiantum reniforme* | LPC18 | Wah013 (IBSC) | KJ742767 | KJ742745 | KJ742788 | KJ779989 | KJ780010 | Cubo dela Galga, La Palma, Canary Islands, Spain |
| *Adiantum reniforme* | MS4 | Wah017 (IBSC) | KJ742761 | KJ742746 | KJ742791 | KJ779978 | KJ780013 | St. Vincent, Madeira, Portugal, |
| *Adiantum reniforme* | MS12 | Wah017 (IBSC) | KJ742759 | KJ742747 | KJ742792 | KJ779979 | KJ780014 | St. Vincent, Madeira, Portugal |
| *Adiantum reniforme* | MS15 | Wah017 (IBSC) | KJ742760 | KJ742748 | KJ742793 | KJ779980 | KJ780015 | St. Vincent, Madeira, Portugal |
| *Adiantum reniforme* | MS24 | Wah017 (IBSC) | KJ742762 | KJ742749 | KJ742794 | KJ779981 | KJ780016 | St. Vincent, Madeira, Portugal |
| *Adiantum reniforme* | MSE1 | Wah018 (IBSC) | KJ742764 | KJ742750 | KJ742795 | KJ779982 | KJ780017 | Sexial, Madeira, Portugal |
| *Adiantum reniforme* | MSE9 | Wah018 (IBSC) | KJ742771 | KJ742751 | KJ742796 | KJ779983 | KJ780018 | Sexia, Madeiral, Portugal |
| *Adiantum reniforme* | MSE15 | Wah018 (IBSC) | KJ742768 | KJ742752 | KJ742797 | KJ779984 | KJ780019 | Sexial, Madeira, Portugal |
| *Adiantum reniforme* | ML21 | Wah016 (IBSC) | KJ742773 | KJ742753 | KJ742790 | KJ779991 | KJ780012 | Lugar de Serra, Madeira, Portugal |
| *Adiantum reniforme* | MP1 | Wah014 (IBSC) | KJ742763 | KJ742754 | KJ742789 | KJ779990 | KJ780011 | Pau Bastiao, Madeira, Portugal |
| *Adiantum nelumboides* | H2 | Wah009 (IBSC) | KJ742757 | KJ742731 | KJ742774 | KJ779969 | KJ779998 | Xinxiang, Wanzhou, Chongqing, China |
| *Adiantum nelumboides* | HS8 | Wah001 (IBSC) | KJ742758 | KJ742736 | KJ742779 | KJ779974 | KJ779997 | Xituo, Shizhu, Chongqing, China |
| *Adiantum roborowski* |  | J.-M. Lu 279 (KUN) | JF935289 | JF935366 | JF937242 | JF980635 | JF980551 | Chongqing, China |
| *Adiantum sinicum* |  | J.-M. Lu 269 (KUN) | JF935300 | JF935378 | JF937253 | JF980646 | JF980562 | Yunnan, China |
| *Adiantum philippense* |  | Y.-X. Zhang 009 (KUN) | JF935299 | JF935376 | JF937252 | JF980644 | JF980561 | Yunnan, China |
| *Vittaria flexuosa* |  | WP1246 (KUN) | JF935357 | JF935441 | JF937314 | JF980703 | JF980625 | Vietnam |
