# Supplementary material for: Revision of series Gravesiana (Adiantum L.) based on morphological characteristics, spores and phylogenetic analyses
Source: PLoS One. 2017 Apr 5;12(4):e0172729. doi: 10.1371/journal.pone.0172729 (PMC5381765; doi:10.1371/journal.pone.0172729)
Supplement: S3 Table — H: Height; LR: Length of pinna stalk; FP: Pinna aligned forms; NP: Number of pinna; S: Pinna size; SP: Pinna shape; M: Pinna margin; NV: Number of veins flabellate at base; TV: Vein tendency near upper margin; NSS: Sori number and shape per pinna; T: Pinna texture; P: Powder-covered or not on the abaxial surface of the pinna. (DOC) [file pone.0172729.s003.doc]

**Original measured values of the morphological characters of series *Gravesiana*. H: Height; LR: Length of pinna stalk; FP: Pinna aligned forms; NP: Number of pinna; S: Pinna size; SP: Pinna shape; M: Pinna margin; NV: Number of veins flabellate at base; TV: Vein tendency near upper margin; NSS: Sori number and shape per pinna; T: Pinna texture; P: Powder-covered or not on the abaxial surface of the pinna.**

| **Index_ID** | **Sequence_ID** | **H (cm)** | **LR(mm)** | **FP** | **NP** | **S** | **SP** | **M** | **NV** | **TV** | **NSS** | **T** | **P** |
| --- | --- | --- | --- | --- | --- | --- | --- | --- | --- | --- | --- | --- | --- |
| A1 | G1-3 | 7.5 | 0.5-1.0 | alternate | 7\7 | 8 × 6.5,  8.5 × 7.5,  7 × 6,  6 × 5 | oblong, etc | entire | 7\6\6\5 | curve near upper margin | 1, reniform | coriaceous | yes |
| A2 | RZ4-3 | 9.2 | 1.0-3.0 | opposite | 7 | 11 × 11,  10 × 11,  11 × 11,  10 × 10,  9 × 9 | subround | entire | 8\8\7\6\5 | straight up to terminal | 1 to many, tranversally linear, truncate at false indusia termination | coriaceous | yes |
| A3 | DB1-1 | 13.8 | 0.5-1.5 | opposite | 8\6 | 9 × 7,  10 × 9,  10 × 8,  7 × 8,  6.5 × 7 | obdeltoid | entire | 10\9\9\8\9 | straight up to terminal | 3, reniform | coriaceous | no |
| A4 | HF1-1 | 7.8 | 0.5-1.0 | opposite | 5\5\4 | 11 × 8.5,  10.5 × 6.5,  9.5 × 8,  7 × 6 | obdeltoid | entire | 7\7\7\7\6 | curve near upper margin | 1, tranversally linear, truncate at false indusia termination | coriaceous | yes |
| A5 | GL2-3 | 15.6 | 1.0-2.0 | opposite | 8 | 14 × 15.5,  13 × 15,  14 × 12 | obdeltoid | entire | 9\8\8 | straight up to terminal | 2, reniform | coriaceous | no |
| A6 | GL3-2 | 17 | 1.0-2.0 | opposite | 6 | 15 × 13.5,  14.5 × 16,  12 × 10 | obdeltoid | entire | 9\9\8 | straight up to terminal | 2, reniform | coriaceous | no |
| A7 | GL1-2 | 14.8 | 1.0-2.0 | opposite | 8 | 11 × 13.5,  15 × 13,  11 × 9 | obdeltoid | entire | 9\10\9\7 | straight up to terminal | 3, reniform | coriaceous | no |
| A8 | DX2-3 | 8.1 | 1.0-3.0 | alternate | 6\5 | 10 × 8,  12 × 8,  11 × 8.5 | oblong, etc | entire | 8\7\6\5\6\6 | curve near upper margin | 1, reniform | coriaceous | yes |
| A9 | MS1-1 | 13.1 | 1.0-4.0 | alternate | 12 | 7 × 8.5,  8 × 7.5,  7 × 6,  7 × 6,  5.5 × 4.5,  4.5 × 3.5 | subround, oblong, etc | entire | 9\9\8\7\6 | curve near upper margin | 1, reniform | coriaceous | yes |
| A10 | HB2-8 | 1.8 | ca. 1.0 | alternate | 3 | 4.0 × 4.5,  5.0 × 3.0,  5.0 × 4.5 | obovate, obdeltoid, subround | dentate | 4\4\4 | curve near upper margin | 1, vertically linear | membranous | no |
| A11 | GGL1-2 | 4.2 | ca. 0.5 | opposite | 3\3 | 11.5 × 7,  12 × 9.5,  11 × 7,  8.5 × 6 | obdeltoid | entire | 6\7\6\6 | curve near upper margin | 1, tranversally linear, truncate at false indusia termination | coriaceous | yes |
| A12 | GSQ1-7 | 6.1 | 1.0-2.5 | alternate | 5\3\3\2 | 9 × 8.5,  8.5 × 7.5,  9 × 7,  6.5 × 5.5 | oblong | entire | 7\8\7\6 | curve near upper margin | 1, reniform | coriaceous | yes |
| A13 | GLM1-1 | 1.65 | 1.0-1.5 | alternate,opposite | 3\3\2\3\3 | 4.5 × 4.5,  4.0 × 3.0,  5.0 × 3.0,  4.5 × 2.5 | subround | entire | 6\4\5\5\4 | curve near upper margin | 1, obicular, sometimes reniform | coriaceous | yes |
| A14 | JYL1-1 | 6.7 | ca. 0.5 | opposite | 12 | 10 × 7.5,  9 × 7,  8 × 6 | obdeltoid or subround | entire | 7\6\6\5 | straight up to terminal | 1 to many, tranversally linear, truncate at false indusia termination | coriaceous | yes |
| A15 | FLG1-10 | 25.9 | 1.0-3.0 | opposite | 15 | 13.5 × 12.5,  12.5 × 11.5,  15 × 14,  15 × 12,  14 × 12,  13 × 9,  11.5 × 7.5 | obdeltoid or subround | entire | 8\7\6\8\5 | straight up to terminal | 2, rarely 1, tranversally linear, truncate at false indusia termination | coriaceous | yes |
| A17 | HCZ1-2 | 19.5 | ca. 3.0 | opposite | 18 | 12 × 10,  13 × 11,  11.5 × 10.5,  10.5 × 9,  10 × 9,  9.5 × 6 | obdeltoid or subround | entire | 8\7\7\6\6\5\4 | straight up to terminal | 1 to 3, tranversally linear, truncate at false indusia termination | coriaceous | yes |
| A18 | GDY1-1 | 2.6 | 0.5-1.0 | alternate | 6\5\3 | 8 × 6.5,  6.5 × 5,  7 × 6,  6 × 4.5,  5.5 × 4.5 | subround, oblong, etc | entire | 7\7\8\6\5 | curve near upper margin | 1, reniform | coriaceous | yes |
| A21 | GRX1-3 | 6.8 | 0.5-1.0 | alternate | 5\6\7 | 6.5 × 6.0,  6 × 5,  7 × 6,  6 × 4 | oblong, etc | entire | 6\6\6\4 | curve near upper margin | 1, reniform | coriaceous | yes |
| A22 | GSW1-5 | 4.7 | 0.5-1.0 | alternate | 5\5\6 | 6.5 × 7,  6 × 6,  7 × 6.5,  6 × 5,  5.5 × 3.5 | subround, oblong, etc | entire | 8\6\6\4 | curve near upper margin | 1, reniform | coriaceous | yes |
| A23 | DX1-1 | 15 | 2.0-3.0 | opposite | 12 | 15 × 14,  15 × 16.5,  15.5 × 14.5,  13 × 17 | obdeltoid | entire | 10\10\9\9 | straight up to terminal | 4, reniform | coriaceous | no |
| A24 | DX1-2 | 17 | 1.0-2.0 | opposite | 8 | 16 × 15,  16.5 × 16,  15 × 12.5,  11 × 10,  12 × 9 | obdeltoid | entire | 8\8\8\7\8 | straight up to terminal | 2, reniform | coriaceous | no |
| A25 | DB1-2 | 2.1\2.0\2.5 | 0.5-1.0 | alternate | 3\3\3 | 6.0 × 4.5,  5.0 × 4.5,  4.5 × 3.0,  5.0 × 3.0,  4.5 × 3.0,  5.5 × 4.5 | obovate, obdeltoid, subround | dentate | 4\4\4\4\4 | curve near upper margin | 1, obocular, tranvesally or vertically linear | membranous | no |
| A27 | HB2-9 | 2.0\1.9 | 0.5-1.0 | alternate | 2\2\2 | 5.0 × 5.0,  5.0 × 4.5,  5.0 × 4.5 | obovate, obdeltoid, subround | dentate | 4\4\4\2 | curve near upper margin | 1, vertically linear | membranous | no |
| A28 | HF1-4 | 1.2\1.0\1.1\0.8\1.6\1.3 | 0.5-1.0 | alternate | 3\2\2\2\2\2 | 3.5 × 3.5,  3.5 × 3.5,  5.0 × 4.5,  4.5 × 3.5,  5.5 × 5.5,  4.0 × 3.5 | obovate, obdeltoid, subround | dentate | 4\4\4\4\4\5 | curve near upper margin | 1, obocular, tranvesally or vertically linear | membranous | no |
| A29 | HF1-5 | 2.1\2.6\2.5 | ca. 1.0 | alternate | 2\2\2 | 5.0 × 4.5,  5.5 × 5.0,  6.0 × 5.0,  5.5 × 4.0 | obovate, obdeltoid, subround | dentate | 4\4\4\4 | curve near upper margin | 1, vertically linear | membranous | no |
| A30 | HF1-2 | 3.5\3.5\4.1 | 0.5-1.0 | alternate | 5\5\4\4 | 5.0 × 3.5,  4.0 × 3.0,  4.5 × 3.0,  3.5 × 2.0 | obovate | entire | 4\3\3\4\2 | curve near upper margin | 1, obicular | membranous | no |
| A31 | HF1-3 | 3.5\3.4 | 0.5-1.0 | alternate | 4\4\3\4 | 6.0 × 4.0,  5.0 × 3.5,  4.0 × 3.0 | obovate | entire | 2\2\2\2 | curve near upper margin | 1, obicular | membranous | no |
| A32 | HMM1-9 | 4.5 | 0-1.0 | alternate | 6\7 | 5.0 × 4.5,  4.5 × 4.0,  4.0 × 3.5 | subround | entire | 4\4\4\5 | curve near upper margin | 1, obicular | coriaceous | no |
| A33 | HMM1-10 | 1.5\1.6\1.7 | 0.5-1.0 | alternate | 3\2\3\2 | 3.5 × 4.0,  3.5 × 4.0,  4.5 × 5.5 | obovate, obdeltoid, subround | dentate | 4\4\4\4 | curve near upper margin | 1, obocular, tranvesally or vertically linear | membranous | no |
| A34 | HMM1-11 | 1.9 | ca. 1.0 | alternate | 3\2 | 3.5 × 4.5,  5.5 × 6.5,  4.5 × 4.5 | obovate, obdeltoid, subround | dentate | 4\4\4\4 | curve near upper margin | 1, obocular, tranvesally or vertically linear | membranous | no |
| A35 | JY1-1 | 1.6\2.5 | 0.5-1.0 | alternate | 5\5\5\4\4 | 3.0 × 2.5,  2.5 × 2.5,  3.5 × 3.0,  3.0 × 2.5,  3.5 × 2.5 | obovate | entire | 4\4\4\3\2\2 | curve near upper margin | 1, obicular | membranous | no |
| A36 | JE1-1 | 2.3\1.6 | 0.1-0.5 | alternate | 2\2\2\3\2 | 5.5 × 4,  5 × 3.5,  5 × 4,  5 × 3.5,  5.5 × 4,  5 × 3 | subround, obvate, oblong | entire | 6\5\4\4\4\4 | curve near upper margin | 1, obicular | coriaceous | no |
| A37 | GLM1-2 | 2.1\1.5\1.8 | 1.0-1.5 | alternate,opposite | 5\5\4 | 5.0 × 3.5,  4.5 × 4.0,  5.0 × 3.5 | subround | entire | 4\4\4 | curve near upper margin | 1, obicular, sometimes reniform | coriaceous | yes |
| A38 | GLM2-2 | 11.3 | 3.0-5.0 | alternate | 6 | 9.5 × 8.5,  9.5 × 7.0,  9 × 7,  9.5 × 7.5,  9 × 10.5 | oblong, etc | entire | 8\8\6\7\6 | curve near upper margin | 1, reniform | coriaceous | yes |
| A39 | GGL1-5 | 3.3 | ca. 1.0 | alternate | 3\4 | 12 × 6,  11 × 6,  10 × 6,  10 × 5.5 | oblong | entire | 8\7\7\6 | curve near upper margin | 1, obicular | coriaceous | yes |
| A40 | GGL1-8 |  | ca. 0.5 | alternate | 6 | 8 × 8.5,  9.5 × 11.5,  9 × 9,  7 × 4 | oblong | entire | 7\9\7\8 | curve near upper margin | 1 or 2, reniform | coriaceous | yes |
| A42 | JE1-2 | 1.9\1.5\1.4\1.65\1.8 | 0.1-0.5 | alternate | 3\3\2\2\4 | 4.5 × 4,  4.5 × 4,  4 × 3,  4 × 2.5,  4.5 × 2.5 | subround, obvate, oblong | entire | 6\5\5\5\4\4 | curve near upper margin | 1, obicular | coriaceous | no |
| A43 | JW1-1 | 6.4\7.8\5.0 | 1.0-4.0 | alternate | 4\4\5\5\4\3 | 9 × 5.5,  8.5 × 4,  7.5 × 4.5,  8.5 × 4,  6.5 × 4,  6 × 3.5 | oblong | entire | 5\5\5\6,5\5\5\4\6 | curve near upper margin | 1, reniform | coriaceous | yes |
| A45 | JW2-5 | 11.5 | ca. 0.5 | opposite | 6\6 | 9 × 7.5,  8.5 × 7,  9 × 7,  7 × 5.5 | obdeltoid | entire | 9\8\7\7 | curve near upper margin | 1, rarely 2, reniform or tranversally linear | coriaceous | yes |
| A47 | JW2-8 | 12.5 | 1.0-6.0 | alternate | 8\8 | 9 × 7,  9 × 7.5,  10 × 7,  10 × 6.5 | oblong, etc | entire | 7\8\8\7\6 | curve near upper margin | 1, reniform | coriaceous | yes |
| A48 | GAS1-4 | 8.6 | ca. 0.5 | opposite | 1\2\3 | 10 × 10,  9 × 6.5,  9 × 7 | obdeltoid | entire | 7\6\6 | curve near upper margin | 1 or 2, reniform or tranversally linear | coriaceous | yes |
| A49 | GJK2-2 | 4.3 | 0.5-1.0 | alternate | 4\3\3 | 8 × 5,  7.5 × 4.5,  8 × 5,  8 × 5.5,  6 × 3.5 | oblong, etc | entire | 8\7\7\6\5 | curve near upper margin | 1, obicular | coriaceous | yes |
| A51 | GSQ1-5 | 3.9 | 1.0-2.5 | alternate | 4\4 | 7 × 5.5,  9 × 5,  8 × 5,  7 × 5 | oblong | entire | 8\7\6\6\6\5 | curve near upper margin | 1, reniform | coriaceous | yes |
| A52 | HB1-2 | 4.5\2.65 | 0.5-1.5 | alternate | 7\6\5 | 6 × 5,  6.5 × 6,  5.5 × 5.5,  5.5 × 5,  3.5 × 3 | subround | entire | 6\5\5\6\4 | curve near upper margin | 1, reniform | coriaceous | yes |
| A53 | SDB1-6 | 5\6\6.5 | 1.0-3.0 | alternate | 4\5\3 | 7 × 5,  5.5 × 4 | subround or oblong | entire | 7\8\7\7\6 | curve near upper margin | 1, tranversally linear, sinus at false indusia termination | coriaceous | yes |
| A54 | SHB1-5 | 4.5 | 1.5-2.0 | alternate | 5 | 8 × 5.5,  7 × 5,  8 × 6,  7.5 × 4.5,  8 × 6 | oblong, etc | entire | 6\6\6\7\6 | curve near upper margin | 1, reniform | coriaceous | yes |
| A55 | SHB1-2 | 8.5 | 1.0-2.0 | alternate | 8\9 | 7.5 × 4.5,  7.5 × 4.0,  6.5 × 5.0,  6.0 × 3.5,  5.0 × 3.5 | oblong, etc | entire | 6\5\6\6\5 | curve near upper margin | 1, reniform | coriaceous | yes |
| A56 | SHB1-4 | 6.7\7.1 | 1.0-2.5 | alternate | 6\6 | 7.5 × 6.0,  7.0 × 6.0,  7.5 × 6.0,  7.0 × 6.5,  7.0 × 6.5 | oblong, etc | entire | 5\6\7\6\6 | curve near upper margin | 1, reniform | coriaceous | yes |
| A57 | SHB1-3 | 5.1\6.2 | 0.5-1.5 | alternate,opposite | 7\7 | 6.0 × 4.0,  8.5 × 5.5,  7.0 × 4.5,  9.0 × 5.5,  7.5 × 5.0 | oblong, etc | entire | 5\5\5\4\4 | curve near upper margin | 1, reniform | coriaceous | yes |
| A58 | SHB1-7 | 3.6 | ca. 0.5 | alternate,opposite | 5\6\7 | 5.0 × 4.0,  5.0 × 3.5,  7.0 × 4.5,  6.0 × 2.0 | subround, oblong, etc | entire | 6\6\6\4 | curve near upper margin | 1, reniform | coriaceous | yes |
| A59 | SDB1-1 | 12.6 | 0.5-3.0 | alternate | 9\8\7 | 8.5 × 11,  10 × 8.5,  11 × 6,  8.5 × 6,  9.5 × 10 | subround or oblong | entire | 9\8\6\6\7 | curve near upper margin | 1, tranversally linear, sinus at false indusia termination | coriaceous | yes |
| A60 | SDB1-2 | 9.9\9.5\9 | 2.0-4.0 | alternate | 6\6\5 | 10 × 8.5,  9 × 7,  9 × 6.5,  7.5 × 6,  7.5 × 5.5 | subround or oblong | entire | 7\7\7\6\5 | curve near upper margin | 1, tranversally linear, sinus at false indusia termination | coriaceous | yes |
| A61 | SJB1-2 | 8.4 | 0.5-4.0 | alternate | 8 | 8.5 × 6.5,  8 × 6.5,  8.5 × 7,  7 × 4 | oblong | entire | 7\5\5\5 | curve near upper margin | 1, reniform | coriaceous | yes |
| A62 | SJB1-3 | 4.4\3.0 | 0.5-1.0 | alternate | 6\7\8 | 4.0 × 3.5,  4.0 × 3.0,  5.0 × 3.5,  4.5 × 4.0,  5.0 × 3.0 | subround | entire | 6\6\5\5\5 | curve near upper margin | 1, reniform | coriaceous | yes |
| A63 | SJB1-4 | 4.5\3.8\5.2 | 0.5-1.0 | alternate | 6\7\8 | 6.0 × 3.5,  6.0 × 5.0,  5.5 × 4.0,  5.5 × 3.5,  5.0 × 4.0 | subround | entire | 5\6\5\6\6 | curve near upper margin | 1, reniform | coriaceous | yes |
| A64 | SJB1-7 | 4.6\5.5 | 0.5-1.5 | alternate | 7\10\7 | 6.5 × 4.0,  5.0 × 5.0,  5.0 × 4.5,  5.5 × 5.0,  4.0 × 3.5 | subround | entire | 7\6\6\6\4 | curve near upper margin | 1, reniform | coriaceous | yes |
| A65 | SDX1-1 | 2.4\2.4\3.3\2.5\1.8 | 1.0-1.5 | alternate | 7\7\7\5\4 | 4.0 × 3.5,  5.0 × 3.5,  4.0 × 4.0,  3.0 × 2.5,  3.0 × 3.0 | subround | entire | 4\4\4\5\4 | curve near upper margin | 1, obicular | coriaceous | yes |
| A66 | SDX1-2 | 3.3\2.1\3.0\2.0 | 0.5-1.5 | alternate | 7\7\8\7 | 4.5 × 3.5,  4.0 × 3.5,  4.0 × 3.5,  4.0 × 3.5,  4.0 × 3.8 | subround | entire | 4\4\4\4\4 | curve near upper margin | 1, obicular | coriaceous | yes |
| A67 | ZD2B1-2 | 4.4\4.0\4.1 | 1.5-2.0 | alternate,opposite | 5\4\4\3 | 9.5 × 5.5,  8.5 × 6.0,  8.5 × 5.5,  10 × 5.5,  9.5 × 5.0 | oblong, etc | entire | 5\5\5\6\4 | curve near upper margin | 1, reniform | coriaceous | yes |
| A68 | ZD2B1-4 | 4.7\5.9\6.9 | 2.0-3.0 | alternate,opposite | 5\10\5 | 6.5 × 5.5,  7.5 × 6.0,  8.5 × 5.5,  9.0 × 7.5 | oblong, etc | entire | 4\4\5\5\6 | curve near upper margin | 1, tranversally linear, truncate at false indusia termination | coriaceous | yes |
| A69 | ZD2B1-6 | 6.4 | 1.0-3.0 | opposite | 6 | 9.5 × 9.5,  10.5 × 7.5,  10 × 6,  10 × 6.5 | oblong | entire | 5\5\4\4\4\4 | straight or curve | 1, tranversally linear, truncate at false indusia termination | coriaceous | no |
| A70 | ZD2B1-8 | 7.9\5.9\5.5 | 0.5-3.0 | opposite | 8\6\6 | 9 × 7,  8.5 × 7.5,  8.5 × 6.5,  9.5 × 7,  8.5 × 6.0,  8.5 × 7.5,  8.5 × 7.0 | oblong | entire | 4\5\5\6\5\7\7 | straight or curve | 1, tranversally linear, truncate at false indusia termination | coriaceous | no |
| A71 | ZD2X1-1 | 4.8\5.0 | 1-3.0 | alternate | 9\9\9\9\7\9 | 4.5 × 4.0,  4.5 × 4.5,  5.0 × 5.0,  5.5 × 5.0,  5.0 × 5.0,  5.0 × 5.0 | subround | entire | 4\4\5\4\5\4\4\5 | curve near upper margin | 1, obicular | coriaceous | yes |
| A72 | ZD2X1-2 | 4.0\3.8\3.7\2.5 | 1.0-2.0 | alternate | 8\7\7\8\7\5\6\7\8 | 4.5 × 4.5,  5.5 × 6.0,  5.5 × 6.0,  5.5 × 5.0,  5.0 × 3.5,  5.0 × 5.0 | subround | entire | 4\4\4\4\4\4\4\4 | curve near upper margin | 1, obicular | coriaceous | yes |
| A73 | ZD2X1-4 | 3.6\3.0\1.4\1.4\1.7\2.0 | 1.0-2.5 | alternate,opposite | 7\6\7\5\7\5\6 | 4.5 × 4.5,  4.5 × 4.5,  3.5 × 3.0,  6.5 × 3.5,  6.0 × 3.5 | subround | entire | 4\4\4\4\4 | curve near upper margin | 1, obicular | coriaceous | yes |
| A74 | ZD1X1-2 | 2.9\2.2\1.4\2.7 | 0.5-1.5 | alternate | 4\2\3\4\5\5\5 | 5.5 × 5.0,  5.0 × 4.5,  5.5 × 3.5,  5.0 × 3.5 | subround | entire | 4\4\4\5 | curve near upper margin | 1, obicular | coriaceous | yes |
| A75 | ZD1X1-3 | 3.4\2.5\3.2 | 1.0-2.5 | alternate | 4\5\6\7\3\4\3\4\3\4\5 | 5.0 × 5.0,  4.5 × 4.5,  4.5 × 4.5,  6.5 × 4.5,  5.0 × 5.0 | subround | entire | 5\4\5\4\4 | curve near upper margin | 1, obicular | coriaceous | yes |
| A76 | ZD1X1-4 | 3.5\4.5\1.7 | 0.5-2.0 | alternate,opposite | 7\5\6\5\7\5, 4\4\3\4\4\3 | 5.0 × 4.0,  5.0 × 4.5,  4.5 × 3.5,  4.0 × 3.5 | subround | entire | 4\4\4\4 | curve near upper margin | 1, obicular, sometimes reniform | coriaceous | yes |
| A77 | ZD1X1-6 | 3\2.9\2.2\2.5 | 2.0-5.0 | alternate | 2\3\3\3\3\5\3, 4\4\3\4\4 | 5.0 × 4.0,  5.0 × 4.5,  5.0 × 4.5 | subround | entire | 4\5\4 | curve near upper margin | 1, obicular | coriaceous | yes |
| A78 | ZD1B1-1 | 4.2\3.5 | 1-2.0 | alternate | 6\6\6 | 8.0 × 6.0,  7.0 × 5.0,  7.0 × 5.5,  5.0 × 4.5,  7.5 × 5.0 | subround, oblong, etc | entire | 6\5\6\6\6 | curve near upper margin | 1, reniform | coriaceous | yes |
| A79 | SFX1-1 | 1\1.6 | ca. 1.0 | alternate | 1\2\2\1\1,4\2\2\3\1 | 4.5 × 4.0,  4.5 × 3.0,  4.5 × 3.5,  4.0 × 2.5 | subround | entire | 4\4\4\4 | curve near upper margin | 1, obicular | coriaceous | yes |
| A80 | SFX1-2 | 1.6\1.0 | ca. 1.0 | alternate | 1\2\2\3 | 5.5 × 4.5,  4.5 × 4.0,  5.0 × 4.0,  4.0 × 3.0 | subround | entire | 4\4\4\4 | curve near upper margin | 1, obicular | coriaceous | yes |
| A81 | SFX1-3 | 1.4\1.6 | 0.5-1.0 | alternate | 2\2\1 | 6.0 × 5.0,  5.0 × 3.0,  4.5 × 3.5,  3.0 × 2.5 | subround | entire | 4\4\4\4 | curve near upper margin | 1, obicular | coriaceous | yes |
| A82 | SHB1-1 | 5.5\6.1\4.6 | 1.0-2.0 | alternate | 5\5\5 | 7.0 × 5.5,  6.5 × 5.0,  7.5 × 7.5,  7.0 × 6.0,  7.0 × 5.5 | oblong,etc | entire | 4\6\6\5\6\7, 4\5\5\6\5 | curve near upper margin | 1, reniform | coriaceous | yes |
| A83 | SS1-1 | 3.7\2.7\2.4 | 1.0-1.5 | alternate | 8\8\8\7\7\5 | 4.0 × 2.5,  3.5 × 2.5,  4.5 × 3.0,  4.5 × 3.0 | subround | entire | 4\4\4\4\4\4 | curve near upper margin | 1, obicular | coriaceous | yes |
| A84 | SS1-4 | 2.2\2.1\1.9 | 0.5-1.5 | alternate | 5\4\5\5,  4\3\3, 3\3\5 | 3.5 × 2.5,  5.0 × 2.5,  4.5 × 3.0 | subround | entire | 2\4\4\4 | curve near upper margin | 1, obicular | coriaceous | yes |
